# Novel Plant-Based Metabolites as Disinfectants against *Acanthamoeba castellanii*

**DOI:** 10.3390/antibiotics11020248

**Published:** 2022-02-14

**Authors:** Ruqaiyyah Siddiqui, Noor Akbar, Bushra Khatoon, Muhammad Kawish, Muhammad Shaiq Ali, Muhammad Raza Shah, Naveed Ahmed Khan

**Affiliations:** 1 College of Arts and Sciences, American University of Sharjah, Sharjah 26666, United Arab Emirates; rsiddiqui@aus.edu (R.S.); noormicrobiologist555@gmail.com (N.A.); 2 H.E.J. Research Institute of Chemistry, International Centre for Chemical and Biological Sciences, University of Karachi, Karachi 75270, Pakistan; bushranaveed57@gmail.com (B.K.); kawishiqbal02@gmail.com (M.K.); shaiq303@hotmail.com (M.S.A.); raza.shah@iccs.edu (M.R.S.); 3 Department of Clinical Sciences, College of Medicine, University of Sharjah, Sharjah 27272, United Arab Emirates

**Keywords:** *Acanthamoeba castellanii*, plant-based natural compounds, betulin, betulinic acid, encystation, excystation, cytotoxicity, cytopathogenicity

## Abstract

Due to global warming, coupled with global water shortages and the reliance of the public on household water tanks, especially in developing countries, it is anticipated that infections caused by free-living amoebae such as *Acanthamoeba* will rise. Thus, the development of novel disinfectant(s) which can target pathogenic free-living amoebae effectively is warranted. Herein, we extracted and isolated several plant-based secondary metabolites as novel disinfectants for use against pathogenic *Acanthamoeba*. The identity of the compounds was confirmed by nuclear magnetic resonance and tested for antiamoebic activities against clinical isolate of *A. castellanii*, belonging to the T4 genotype. Amoebicidal assays revealed that the compounds tested showed antiamoebic properties. Betulinic acid and betulin exhibited parasite killing of more than 65%. When tested against the cyst stage, betulinic acid, betulin, and vanillic acid inhibited both encystation and excystation processes. Furthermore, the plant-based metabolites significantly inhibited the binding capability of *A. castellanii* to host cells. Finally, most of the tested compounds displayed minimal cytotoxic activities against human cells and noticeably perturbed amoeba-mediated host cell cytotoxicity. Notably, both alkaloid and betulinic acid showed 20% cytotoxic effects, whereas betulin and lupeol had cytotoxic effects of 24% and 30%, respectively. Overall, our findings indicate that plant-based natural compounds demonstrate anti-Acanthamoebic properties, and they have potential candidates for water disinfectants or contact lens disinfecting solutions, as well as possible therapeutic drugs against *Acanthamoeba* infections.

## 1. Introduction

*Acanthamoeba* keratitis (AK) is a sight-threatening infection of the cornea, while granulomatous amoebic encephalitis (GAE) is a devastating infection of the brain [1,2,3,4]. The causative agent, *Acanthamoeba*, alternates between two stages—namely, the infective trophozoite stage and the hardy double-walled cyst stage. With an increase in global warming, coupled with global water shortages and public reliance on household water tanks, especially in developing countries, it is likely that infections caused by free-living amoebae will rise; thus, the development of novel disinfectants which can target amoebae effectively is needed [5]. Furthermore, in the case of AK infection, because of the presence of amoebae cysts, recurrent amoebic infections are observed, as the cysts may withstand available therapies and revert to the active trophozoite stage following the treatment [4,6,7,8]. Antimicrobial topical treatments are available for AK infection and usually comprise a combination of propamidine isethionate and neomycin or chlorhexidine, while contact lens disinfectants often include chlorhexidine [2]. Notably, a plethora of studies have shown that many of the contact lens disinfectants commercially available in the market are ineffective against *Acanthamoeba* [9,10], suggesting the need to identify the compound(s) that are biologically relevant, cost effective, effective against cysts and trophozoites, and nontoxic, and which can be used as disinfectants and/or therapeutic agents [11,12,13,14,15,16].

In this regard, natural products have been a major source for innovative drugs, and/or being useful ‘lead’ molecules that can be modified further during the drug development process [17]. The various architectures and complicated carbon skeletons of natural resources have resulted in a substantial fraction of natural products being used in drug discovery. Secondary metabolites derived from natural sources are frequently regarded as having greater ‘drug-likeness and biologically friendly’ than completely synthetic compounds, making them promising candidates for disinfectants, contact lens disinfectants, and drug development [18]. Medicinal plants are rich sources of antimicrobial secondary metabolites [19,20,21,22]. Antiparasitic plant extracts or secondary metabolites generated from them provide an alternative to manufactured medications [19]. For example, previous studies have shown that betulinic acid has antitumor pharmacological effects through apoptosis utilising the mitochondrial pathway, as well as antiviral, antibacterial, antihelmintic activities, and anti-inflammatory effects [23,24]. Other studies have shown that vanillic acid, another plant derivative, has antimicrobial properties [25]. In the current study, we utilised two medicinal plants—namely, *Rinorea yaundensis* and *Salvia triloba*. Furthermore, we accomplished phytochemical studies, resulting in the isolation and characterisation of various secondary metabolites, including a new monoterpenoid indole alkaloid, yaundentine hydrochloride, which was shown to exhibit antimicrobial, antioxidant, urease, and lipoxygenase inhibitory activities [26]. Herein, we tested yaundentine hydrochloride, methyl *β*-orcinol carboxylate, ursolic acid, and betulinic acid against *A. castellanii* for their antiamoebic activities.

## 2. Materials and Methods

### 2.1. Collection and Identification of Plants

The aerial parts of *R. yaundensis* and leaves of *S. triloba* were collected during the month of April and May. The former plant was obtained from Batouri, Bertoua, and East Cameroon, particularly from the ALOUMBOUL forest around SAKOUA River, and identified by Mr. Nana Victor at the National Herbarium of Yaoundé Cameroon. The latter is from Al-Khalil (Southern Palestine) and was identified by Prof. Dawood Al-Asawi, Department of Biological Sciences, University of Jordan, Amman.

### 2.2. Extraction and Isolation

The aerial parts of *R. yaundensis* (27 kg) and leaves of *S. triloba* (9 kg) were shade dried and extracted thrice with methanol (3 × 30 L), at room temperature. The combined methanolic extracts of both the plants were freed of solvent at room temperature on a thin film rotary evaporator, to obtain crude extracts (405.6 g from *R. yaundensis* and 550 g from *S. triloba*). The crude extract from *R. yaundensis* was chromatographed over silica gel and eluted with mixtures of n-hexane, DCM–hexane, DCM, and DCM–MeOH in increasing order of polarity. The fractions which eluted with DCM–hexane (50–70%) provided methyl *β*-orinol carboxylate (70 mg). The fraction which eluted with DCM was rechromatographed and eluted with mixtures of hexane–ethyl acetate. The fraction which eluted with hexane–ethyl acetate (95:5) furnished betulin acid. The remaining fractions which eluted with DCM–MeOH (85:5) to 100% MeOH were combined and divided into ethyl acetate soluble and insoluble fractions. The latter was subjected to flash chromatography. Elution with hexane–EtOAc (90% to 95%) provided yaundentine hydrochloride (500 mg). On the other hand, the ethyl acetate soluble fraction was chromatographed over silica gel. Elution with DCM provided a major compound, along with lingering traces of impurities. Further chromatography and elution with hexane–ethyl acetate (90:10) resulted in the isolation of pure ursolic acid. 

The crude extract of *S.triloba* was chromatographed over silica gel and eluted with mixtures of hexane–ethyl acetate and ethyl acetate–methanol in increasing order of polarity. The eluate obtained with hexane–ethyl acetate (70:30) was triturated with methanol, to obtain pure crystals of betulin (700 mg). The fraction eluted with hexane–ethyl acetate (60:40) furnished oleanolic acid (245 mg). Further increase in polarity (50–60%) of ethylacetate led to the isolation of vanillic acid (862 g). The fraction eluted with ethyl acetate–methanol (5–20%) comprised a major compound which was purified by further chromatography and elution with 40–70% ethylacetate–hexane, leading to the isolation of rosmarinic acid (650 mg). 

The purity of all the isolates was checked with the help of TLC and HPLC, respectively. 

### 2.3. Characterisation of Secondary Metabolites Extracted from R. yaundensis and S. triloba

Yaundentine hydrochloride amorphous powder; mp 309–318 °C; ${\left[\alpha \right]}_{D}^{25}$ 37.92° (*c* 7 × 10^−3^, MeOH); UV (MeOH) λ_max_ (log *ɛ*) 229 (2.19), 242 (2.12), 286 (1.74) nm; IR (KBr) v_max_ 3396, 2978, 1591, 1480 and 1299 cm^−1^; CD (*c* 7 × 10^−3^, MeOH) Δ*ɛ* (λ nm): −3.5 (442), +2.24 (428), −2.65 (402), +1.65 (388), −1.21 (364), +1.08 (344), −15.23 (290), +19.6 (246), −1.86 (228), +8.72 (212), +4.24 (208); ^1^H (CD_3_OD, 400 MHz) *δ* 7.03 (1H, br d, *J* = 7.1, H-9), 6.63 (1H, t, *J* = 7.1, H-10), 6.61 (1H, t, *J* = 7.1, H-11), 5.54 (1H, q, *J* = 6.4, H19), 4.44 (1H, s, H-17), 3.99 (1H, br.d, *J* = 14, H-3), 3.97 (1H, br.d, *J* = 14, H-21a), 3.86 (1H, br.d, *J* = 14, H-21b), 3.68 (1H, br.t, *J* = 6.1, H-5), 3.46 (1H, s, H-2), 3.40 (1H, t, *J* = 6.1, H-15), 2.46 (1H, t, *J* = 6.1, H-16), 2.23 (1H, br.d, *J* = 13.6, H-6b), 2.15 (1H, m, H-6a), 2.15 (2H, m, H-14), 1.76 (3H, d, *J* = 6.4, H-18).

Methyl *β*-orcinol carboxylate white crystal; m.p 142–144 °C; UV λ_max_ (MeOH) (logε) 221 (2.20), 237 (2.75), and 319 (3.01) nm; IR (KBr) v_max_ 3595, 1690, and 1600 cm^−1^; EI-MS *m/z* 196 [M⁺]; ^1^H (CD_3_OD, 400 MHz) *ẟ* 6.20 (1H, s, H-5), 3.88 (3H, s, OCH_3_), 2.41 (3H, s, CH_3_-6), and 1.99 (3H, s, CH_3_-3).

Betulinic acid white powder; m.p 279–281 °C; ${\left[\alpha \right]}_{D}^{25}$ +8.1° (*c* 9.1 in CH_3_OH); IR (KBr) v_max_ 3250, 1750, and 1640 cm^−1^; EI-MS *m/z* 456 [M⁺]; ^1^H (C_5_D_5_N, 400 MHz) *ẟ* 4.93 (1H, s, H-29a), 4.76 (1H, s, H-29b), 3.58 (1H, m, H-19), 3.47 (1H, m), 1.78, 1.19, 1.03, 1.02, 0.977, and 0.79 (3H, s, 6 × CH_3_).

Ursolic acid white powder; m.p 277–280 °C; ${\left[\alpha \right]}_{D}^{25}$ +60.9° (*c* 0.9, CHCl_3_); IR (KBr) v_max_ 3300, 1745 and 1640 cm^−1^; EI-MS *m/z* 456 [M⁺]; ^1^H (C_5_D_5_N, 400 MHz) *ẟ* 5.22 (1H, t, *J* = 4.0 Hz, H-12), 3.46 (1H, t, *J* = 7.44 Hz, H-3), 0.96 (3H, d, *J* = 5.7 Hz, H-29), 0.88 (3H, d, *J* = 6.4 Hz, H-30), 1.22, 1.20, 0.95 0.84, and 0.86 (3H, s, 5 × CH_3_).

Oleanolic acid white powder; m.p 271–276 °C; ${\left[\alpha \right]}_{D}^{24}$ +65° (*c* 0.30, CHCl_3_), IR (KBr) v_max_ 3433, 1690 and 1641 cm^−1^; EI-MS *m/z* 456 [M^+^]; ^1^H-NMR (C_5_D_5_N, 400 MHz) *ẟ* 5.49 (1H, br. t, H-12), 3.29 (1H, dd, *J* = 2.56, 9.1 Hz, H-3), 1.27, 1.23, 1.02, 1.02, 1.00, 0.94 and 0.88. (3H, s, 7 × CH_3_).

Betulin amorphous white powder; m.p 244–246 °C; ${\left[\alpha \right]}_{D}^{26}$ +12 (*c* 0.399, CHCl_3_); IR (KBr) v_max_ 3410, 1645, 1030 cm^−1^; ^1^H (CDCl_3_, 400 MHz) *ẟ* 4.65 (1H, br s, H-29a), 4.56 (1H, br d, H-29b), 3.79 (1H, d, *J* = 10.5 Hz, H-28a), 3.32 (1H, d, *J* = 10.8 Hz, H-28b), 1.73, 1.19, 0.97, 0.96, 0.82, and 0.78 (3H, s, 6 × CH_3_).

Vanillic acid off-white powder; m.p 210–214 °C; UV λmax (MeOH) 279 nm; IR (KBr): 3433, 1691, 1635 cm^−1^; EI-MS: *m*/*z* 168 [M^+^]; ^1^H (CD_3_OD, 400 MHz): *ẟ* 7.55 (1H, dd, *J* = 2, 6.6 Hz, H-6), 7.54 (1H, d, *J* = 2 Hz, H-2), 6.83 (1H, d, *J* = 8.7 Hz, H-5) and 3.88 (3H, s, OCH_3_).

Rosmarinic acid white powder; m.p 171–176 °C; UV λmax (MeOH) 254, 270 nm; IR (KBr): 3400, 1698, 1645 cm^−1^; ^1^H (CD_3_OD, 400 MHz): *ẟ* 7.55 (1H, d, *J* = 15.9 Hz, H-7), 7.03 (1H, d, *J* = 1.9 Hz, H-2), 6.95 (1H, dd, *J* = 1.9, 8.2 Hz, H-6) and 6.77 (1H, d, *J* = 8.1 Hz, H-5) 6.74 (1H, d, *J* = 1.9, H-13), 6.69 (1H, d, *J* = 8.04 Hz, H-16), 6.61 (1H, dd, *J* = 1.9, 8.08 Hz, H-17), 6.27 (1H, d, *J* = 15.8 Hz, H-8), 5.19 (1H, dd, *J* = 4.3, 8.3 Hz, H-10), 3.11 (1H, m, H-11a), 2.96 (1H, m, H-11b).

### 2.4. Acanthamoeba castellanii Cultures

*A. castellanii* genotype T4 (ATCC 50492) were cultivated in 10 mL of protease-peptone–yeast–glucose (PYG) broth medium (0.75% yeast extract, 0.75% protease-peptone, and 1.5% glucose). When the amoebae cultures attained confluency, the cultured flask was placed on ice for 10 min before gently tapping to detach the adherent trophozoites. After centrifuging the amoebae culture for 5 min at 2500× *g*, the pellet was resuspended in 1 mL of Roswell Park Memorial Institute (RPMI) media after discarding the supernatant. Subsequently, the number of *A. castellanii* was assessed using a haemocytometer. The starting *A. castellanii* inoculum (5 × 10^5^) was adjusted by enumerating with a haemocytometer and then used for several tests [12,13].

### 2.5. Amoebicidal Assays

*A. castellanii* were treated with the compounds isolated from medicinal plants to investigate their antiamoebic effects, as described previously [27,28]. Briefly, in a 24-well plate, amoebae (5 × 10^5^) were challenged with plant-based natural compounds (100 µg/mL) at 30 °C, for 24 h, with a final assays volume of 0.5 mL. Amoebae cultured in RPMI alone was taken as a negative control, and 0.25 percent sodium dodecyl sulphate (SDS) was used as a positive control. Finally, 0.1% Trypan blue was added to each well, and viable amoebae trophozoites were counted using a haemocytometer [12,13].

### 2.6. Henrietta Lacks Cervical Adenocarcinoma (HeLa) Cell Lines Cultivation

HeLa cells were obtained from the American Type Culture Collection (ATCC CCL-2) and were cultured in RPMI supplemented, with 1% minimum essential medium amino acids, 10% fetal bovine serum (FBS), 1% *L*-glutamine, and 1% penicillin–streptomycin (Pen-Strep) at 37 °C, in a humidified condition, with 5% CO_2_. Upon this incubation, the media were removed, and adherent cells were enzymatically disengaged with 2 mL trypsin EDTA, followed by 5 min centrifugation at 2500× *g*. The cells were resuspended in the abovementioned complete media and seeded into 96-well plates that were used in several assays [29].

### 2.7. Adhesion Assays

Adhesion experiments were used to determine how plant-based natural compounds affected amoebae binding to human cells. Briefly, 5 × 10^5^ amoeba trophozoites were cultured for 2 h in serum-free RPMI-1640 medium at 30 °C with plant-based chemicals (100 µg/mL). The pretreated amoebae were then centrifuged for 5 min at 2500× *g* and resuspended in 200 µL of RPMI-1640. The entire assay volume containing pretreated amoeba was transferred to HeLa cell monolayers grown in 96-well plates, and the plates were incubated for 1 h at 37 °C, with 5% CO_2_ and humidified conditions. The unbound amoebae were then enumerated using a haemocytometer, and the percent bound amoebae was determined using the following formula: percent amoebae (bound) = 100 − amoebae (unbound). As a control, untreated amoebae were cultivated with HeLa cell monolayers [29].

### 2.8. Encystation Assays

Plant-based natural compounds were evaluated against *A. castellanii*, to determine their effects on the encystation process [30]. In brief, one million amoeba trophozoites were incubated with plant-based natural compounds in the presence of 16% filter-sterilised glucose (final concentration) for 48 h at 30 °C. Next, SDS (0.1%) was added to each well of the 24-well plate, which was agitated for 20 min. The remaining cysts were counted, and the data were recorded using a haemocytometer. *A. castellanii* were cultured alone in 16% glucose as a control [31].

### 2.9. Excystation Assays

*A. castellanii* cysts were prepared by growing 3 mL of amoeba culture suspended in phosphate-buffered saline (PBS) on non-nutrient bacteriological agar plates at 30 °C for two weeks [32]. Concisely, PBS was applied to non-nutrient agar plates (which contained the amoeba cysts), and amoebae cysts were scraped off the plates. To adjust the starter culture for the experiments, the amoebae culture was centrifuged at 3000× *g* for 10 min and the pellet was resuspended in serum-free RPMI. To investigate the excystation process, 1 × 10^5^ *A. castellanii* cysts were incubated in PYG medium with 100 µg/mL of plant-based natural compounds (final volume 500 µL). Amoeba cysts grown in PYG alone were taken as a control. The plates were incubated and observed on regular basis for 24–72 h at 30 °C. Finally, the viable amoebae trophozoites were calculated, and the data were recorded using a haemocytometer [33].

### 2.10. In Vitro Cytotoxicity Assays

Lactate dehydrogenase (LDH) assays were performed, to determine the *in vitro* cell cytotoxicity of plant-based natural compounds using human cells [34,35]. Briefly, the HeLa cells monolayer was challenged with 100 µg/mL plant-based natural compounds in a 96-well plate. The plate was incubated at 37 °C, with 95% humidity and 5% CO_2_, for 24 h. Next, triton X-100 (0.1%) was added to the positive control wells and incubated the plate at 37 °C for 45 min. Subsequently, an equal volume of LDH kit reagents (Cytotoxicity Detection kit; Roche Diagnostics, Indianapolis, IN, USA), was mixed with equal cell supernatant containing liberated LDH enzyme, to assess the LDH released as follows: % cytotoxicity = sample value – negative control value/positive control value – negative control value × 100. Cell monolayer incubated with 0.1% Triton X-100 and in RPMI alone were taken as positive and negative controls, respectively [36].

### 2.11. Amoeba-Mediated Host Cell Death

To determine the amoeba-mediated host cell death, cytopathogenicity tests were performed as previously described [28,30]. To summarise, plant-based natural compounds at a final concentration of 100 µg/mL were incubated with amoebae (5 × 10^5^) for 120 min, at 30 °C. Upon this incubation, the pretreated amoebae were centrifuged for 5 min at 2500× *g*, and the cell pellet was resuspended in 200 µL of serum-free RPMI. The total assay volume containing pretreated amoebae was subjected to a confluent HeLa cell monolayer grown overnight in 96-well plates. The plates were incubated at 37 °C, with 5% CO_2_ and 95% humidity, for 24 h. Lastly, amoeba-mediated host cell death was measured indirectly by assessing the amount of LDH enzyme released into cell media by damaged cells, as previously reported [34,35]. 

### 2.12. Statistical Analyses

All statistical comparisons were conducted using a two-sample *t*-test, two-tailed distribution. The data are presented as the mean ± standard error of several replicated studies. GraphPad Prism version 8.0.2 was used for all of the analyses and visualisations (GraphPad Software; San Diego, CA, USA). *p* ≤ 0.05 was considered the statistical significance level [35].

## 3. Results

### 3.1. Plant-Based Natural Compounds Isolated from R. yaundensis and S. triloba

The results from nuclear magnetic resonance (NMR) revealed 10 plant-based natural compounds (Figure 1). The NMR spectra confirmed their presence in the crude extracts (Supplementary Figures S1–S27).

### 3.2. Plant-Based Natural Compounds Presented Effective Amoebicidal Activity against A. castellanii

**Figure 2:**
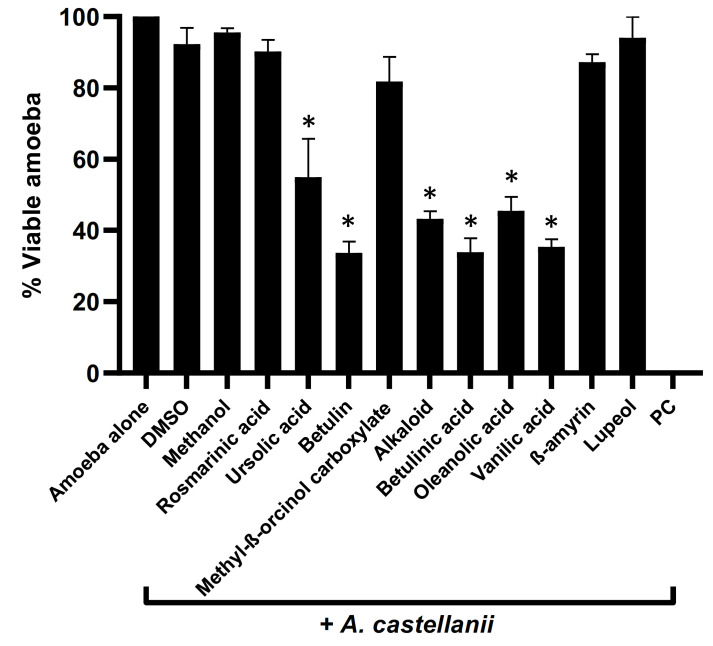
Compounds isolated from medicinal plants presented notable amoebicidal activities against amoeba. Briefly, isolated compounds at a concentration of 100 µg/mL were incubated with 5 × 10^5^ of *A. castellanii* overnight, at 30 °C. Following this, viable amoebae were calculated using haemocytometer microscopically. The data are expressed as the mean ± standard error. *p* values were determined using two-sample *t*-test, two-tailed distribution, (*) is *p* < 0.05.

Plant-based natural compounds were evaluated to determine their amoebicidal activity against *A. castellanii*. The results showed that all the tested natural compounds except rosmarinic acid, methyl β-orcinol carboxylate, β-amyrin, and Lupeol exhibited potent amoebicidal effects against *A. castellanii* (*p* < 0.05, two-tailed distribution) (Figure 2). Among all compounds tested, betulin, betulinic acid, and vanillic acid presented 67%, 67%, and 65% activities against *A. castellanii* (Figure 2). Similarly, a decrease of 57%, 55%, and 45% in amoeba viability was observed for alkaloid, oleanolic acid, and ursolic acid, respectively.

### 3.3. Natural Compounds Tested Blocked Amoebae Binding to Human Cells

**Figure 3:**
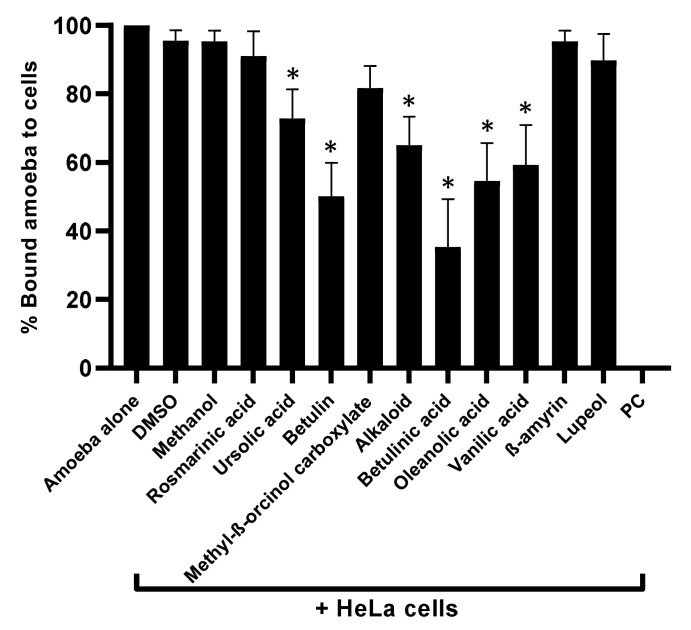
Compounds significantly blocked the *A. castellanii* binding to human cells. Adhesion assays were carried out to examine whether *A. castellanii* interact with human cells. *p* values were determined using two-sample *t*-test, two-tailed distribution, (*) is < 0.05. The data are represented as the mean ± standard error of three independent experiments performed in duplicate.

Plant-based natural metabolites were tested for their capability to block amoebae binding to human cells, using adhesion assays. The results revealed that all the drugs with amoebicidal activity significantly repressed the binding capabilities of *A. castellanii* to HeLa cells (*p* < 0.05) (Figure 3). Parallel to amoebicidal effects, betulinic acid showed the highest activity inhibited 65% of amoeba binding to human cells (Figure 3). Betulin showed 50% inhibition, while oleanolic acid and vanillic acid inhibited amoebae binding to human cells up to 46% and 41%, respectively. Alkaloid and ursolic acid exhibited 35% and 27% amoeba blocking ability to host cells. The remaining compounds failed to inhibit amoebae binding to the human cell monolayer (Figure 3).

### 3.4. Plant-Based Secondary Metabolites Considerably Inhibited Amoebae Encystation and Excystation

**Figure 4:**
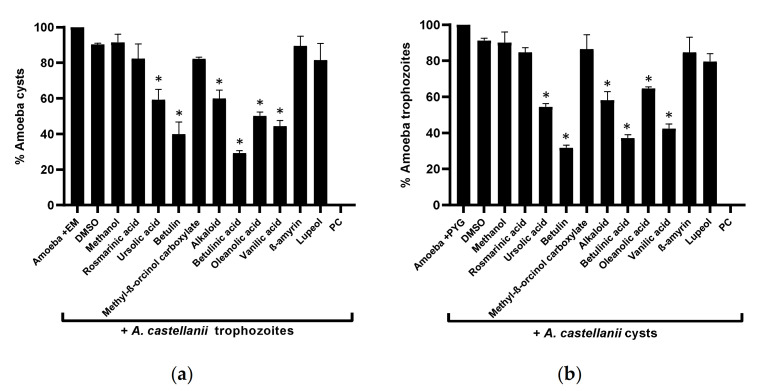
Effects of isolated compounds on the encystation and excystation of *A. castellanii*. The results revealed that compounds inhibited both encystment and excystment processes in *A. castellanii*, compared with the negative control: (**a**) the encystation process; (**b**) the excystation effects. The data are presented as the mean ± standard error. *p* values were calculated using a two-sample *t*-test with two-tailed distribution; (*) denotes that *p* < 0.05.

The overall results revealed that the plant-based secondary metabolites successfully inhibited the encystation process in *A. castellanii* when compared with the negative control (*p* < 0.05) (Figure 4a). Betulinic acid showed the highest activity, and the number of cysts formation drastically declined to 29%, compared with the negative control (100%). Similarly, betulin, vanillic acid, and oleanolic acid arrested amoeba encystation dropped to 40%, 44%, and 50%, respectively, while alkaloid and ursolic acid prevented 41% and 40% amoeba encystment. For the excystment assays, a similar pattern of activity to encystation was observed—namely, that significantly inhibited *A. castellanii* cysts re-emerge as viable trophozoites (*p* < 0.05) (Figure 4b).

### 3.5. Natural Compounds Tested Showed Marginal Cytotoxic Properties against Human Cell Lines and Reduced Amoebae-Mediated Host Cell Death

**Figure 5:**
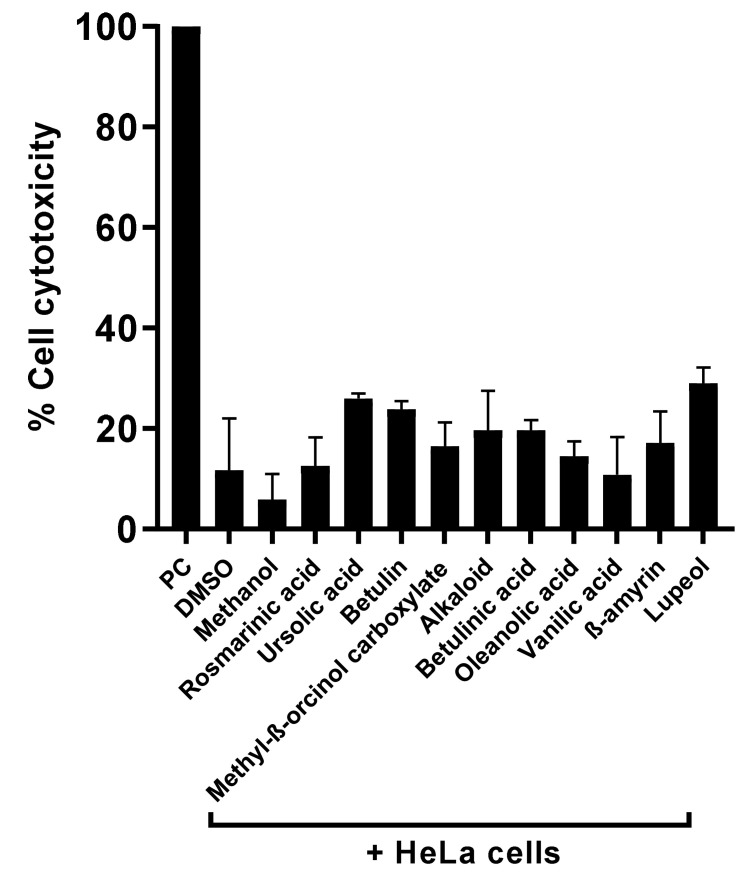
Compounds isolated presented higher cell viability effects against human cell lines. Notably, all compounds showed negligible cytotoxic effects against HeLa cell lines.

**Figure 6:**
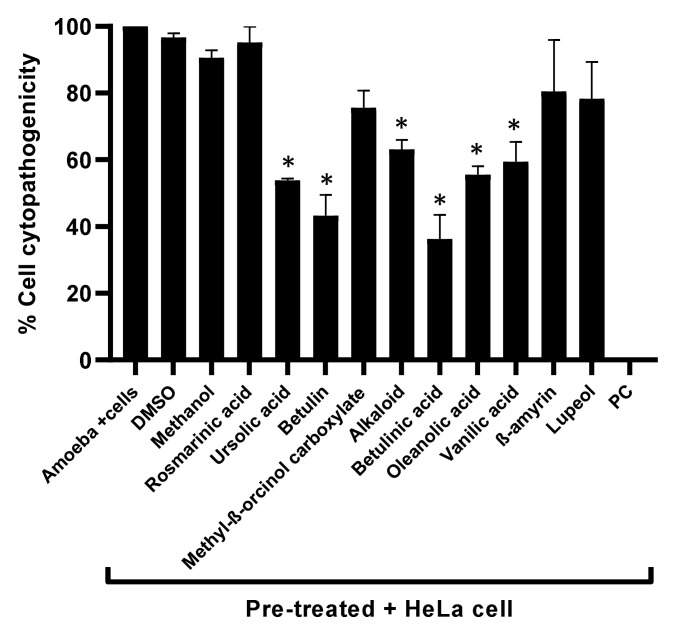
The cytopathogenicity of human cells was declined by pretreating *A. castellanii* with compounds. The results revealed considerable inhibition of amoeba-mediated host cytotoxicity when compared with amoeba (untreated). Data are presented as mean ±  standard error. *p* values were calculated using a two-sample *t*-test with two-tailed distribution, and (*) denotes that *p* < 0.05.

To measure the cytotoxic effects of the plant-based secondary metabolites towards human cells, lactate dehydrogenase assays were accomplished. Results from cytotoxicity assays revealed that most of the tested plant-based natural compounds offered negligible cell cytotoxic activity against HeLa cell lines (Figure 5). Notably, both alkaloid and betulinic acid showed 20% cytotoxic effects, whereas betulin and lupeol had cytotoxic effects of 24% and 30%, respectively. All other compounds revealed cytotoxic effects of less than 20% (Figure 5). In some experiments, amoeba-mediated host cell cytopathogenicity was performed, in which *A. castellanii* was pretreated with all test compounds before being introduced into a confluent HeLa cells monolayer. The results indicated that all drugs with significant amoebicidal activities significantly abridged amoebae-mediated host cell cytotoxicity, compared with the negative control (Figure 6). Betulinic acid and betulin showed the maximum effects and reduced the host cell death up to 36% and 43%, respectively (Figure 6).

## 4. Discussion

*Acanthamoeba* is a unicellular free-living protist that is found in a wide range of environments and causes a serious eye infection, as well as a fatal infection of the central nervous system (CNS) [37,38,39,40]. Troublingly, parasites, in general, including waterborne parasites, are responsible for significant morbidity and mortality [41]. With the escalation in global warming and increasing global water shortages, it is likely that infections due to waterborne pathogens including free-living amoebae will rise, with many members of the public resorting to private storage tanks to house water, especially in the developing world [5,42]. Medicinal chemists have introduced a number of compounds that may be used to treat a variety of endoparasites; however, there are a limited number of compounds which are efficacious against free-living amoeba infections, for use as disinfectants or as therapy, due to factors such as the inability of the drugs to cross the blood–brain barrier, as well as the robust nature of the cyst stage of these amoebae and its ability to endure harsh conditions [12,13]. Furthermore, the pharmaceutical industry has not placed a high priority on the development of new antiparasitic treatments, as many parasitic infections occur in underdeveloped countries. Moreover, due to the rare nature of infections caused by free-living amoebae, as well as the lack of awareness amongst clinicians, the true burden of infections due to free-living amoebae is not known [14]. As a result, investing in antiparasitic disease drug development is not well rewarded [19].

Significant attention has been given over the years to the production of potent antimicrobial secondary metabolites [41,43]. Among these, about 900 species of genus *Salvia* are cultivated throughout the world. Most of these species have therapeutic importance and have been used extensively in traditional medicines [44]. *S. triloba* has been used to treat wounds in traditional medicine and is also thought to possess antiseptic, antipyretic, and diuretic activities; thus, it may also possess antiamoebic activities which is the subject of this study [45]. Phytochemical investigations of these medicinal plants in this study yielded 10 secondary metabolites, comprising the novel monoterpenoid indole alkaloid (yaundentine hydrochloride), rosmarinic acid, vanillic acid, botulin, and oleanolic acid. These compounds were evaluated for their antiamoebic activities against *A. castellanii,* as well as encystment and excystment capabilities, for their potential use as disinfectants. Finally, they were tested for their cytopathic effects against human cell lines. The results revealed that out of the 10 compounds tested, 6 presented significant antiamoebic activities against pathogenic *Acanthamoeba*. Among all of the compounds tested, betulinic acid and betulin showed the highest antiamoebic effects. Previously, it has been shown that betulinic acid synergistically enhanced the antimicrobial effects of cystamine and its amides against bacterial and fungal species [46]. Similarly, betulin and betulinic acid have a wide range of therapeutic activities, including anti-inflammatory, antiviral, antibacterial, antidiabetic, and antiprotozoal effects, thus explaining their efficacy against free-living amoebae infections. The effects of betulinic acid and betulin need to be evaluated against other free-living amoebae as well (e.g., *Naegleria fowleri* and *Balamuthia mandrillaris*), so a combination therapy can be developed against various amoebal pathogens [47,48]. 

Of significance is that the compounds betulinic acid, betulin, vanillic acid, alkaloid, oleanolic acid, and ursolic acid blocked encystation and excystation in amoebae, coupled with minimal cytotoxic properties towards human cells, which is very encouraging and indicates the potential of these as novel disinfectants against free-living amoebae infections. Notably, our study revealed that both the alkaloid and betulinic acid showed 20% cytotoxic effects, whereas betulin and lupeol had cytotoxic effects of 24% and 30%, respectively. Previous studies have shown that kolavenic acid and quercetin isolated from the crude extract of *Polyalthia longifolia var pendula* and *Caesalpinia pulcherrima* presented anti-Acanthamoebic activities [30]. Furthermore, amoebicidal activities have been observed in secondary metabolites derived from a number of plants. These include *Origanum syriacum, Arachis hypogaea*, *Allium sativum, Origanum laevigatum, Pancratium maritimum L., Pterocaulon polystarchyum, Croton ericoides, Croton isabelli, Croton pallidulus, Curcuma longa L*. Plant-based natural compounds showed antiamoebic properties against both the infective trophozoite and cyst stages; however, toxicity effects on human cells may vary [49,50,51]. The isolated compounds from *R. yaundensis* and *S. triloba* showed potent antiparasitic effects with minimal cytotoxicity and reduced amoeba mediated host cell death against human cell lines, in comparison with a study whereby kolavenic acid and quercetin isolated from *Polyalthia longifolia* revealed some cytotoxic effects against human cells [30]; additionally, betulinic acid has been shown to have cytotoxic effects against HeLa cell lines. The medicinal plants tested herein were effective while showing some or limited toxicity against human cells, indicating their potential to be developed and used as disinfectants or contact lens disinfectants; however, future research can focus on reducing the toxicity via nanotechnology or other means, such as the use of liposomes.

Another medicinal plant compound tested in this study is the novel monoterpenoid indole alkaloid—yaundentine hydrochloride—which was evaluated here for the first time against *Acanthamoeba*; it presented 57% antiamoebic properties against *A. castellanii*. The alkaloid presented antibacterial, antiurease, antioxidant, and lipoxygenase inhibitory properties [26]. Previous research has shown that alkaloids have been fundamental in the development of a number of antibiotics with various modes of action [52,53]. Additionally, alkaloids isolated from *Yemeni lawsoniainermis* L. have shown promising antibacterial effects against several pathogenic bacteria; thus, this compound is a valuable, prospective candidate as a disinfectant, compared with *Acanthamoeba* and other free-living amoebae of interest [54].

## 5. Conclusions

In conclusion, in the current study, we isolated plant-based secondary metabolites and identified several with potent antiamoebic activities which are good candidates as novel disinfectants or for inclusion in contact lens disinfectants. The next steps will be to test these medicinal plant compounds in animal models of AK or GAE, by injecting intravenously to observe their antiparasitic activity, as well as investigating their capabilities as disinfectants for use in household water storage tanks or inclusion in novel contact lens disinfectants, which will determine the translational value of these very promising findings.

## Figures and Tables

**Figure 1:**
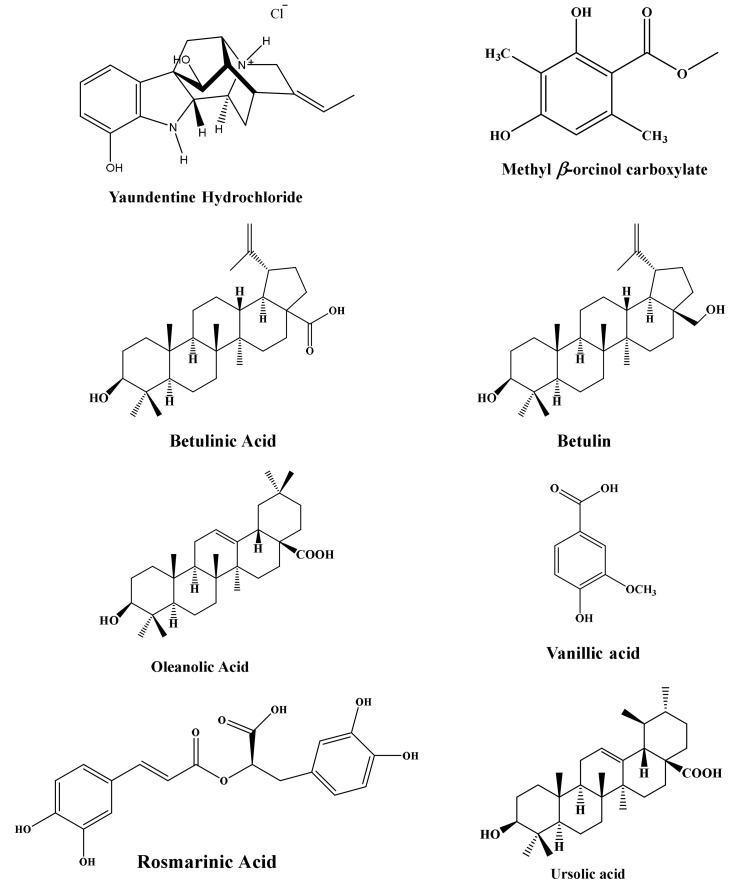
Compounds and their structures isolated from medicinal plants.

## Data Availability

The data presented in this study are available on request from the corresponding author.

## Supplementary Materials

The following supporting information can be downloaded at: https://www.mdpi.com/article/10.3390/antibiotics11020248/s1. The nuclear magnetic resonance (NMR) spectra of plant-based natural compounds tested (Figures S1–S27).
