# Supplementary material for: Novel Plant-Based Metabolites as Disinfectants against Acanthamoeba castellanii
Source: Antibiotics (Basel). 2022 Feb 14;11(2):248. doi: 10.3390/antibiotics11020248 (PMC8868186; doi:10.3390/antibiotics11020248)
Supplement: Supplementary file 1 [file antibiotics-11-00248-s001.zip › antibiotics-1531122-supplementary.pdf]

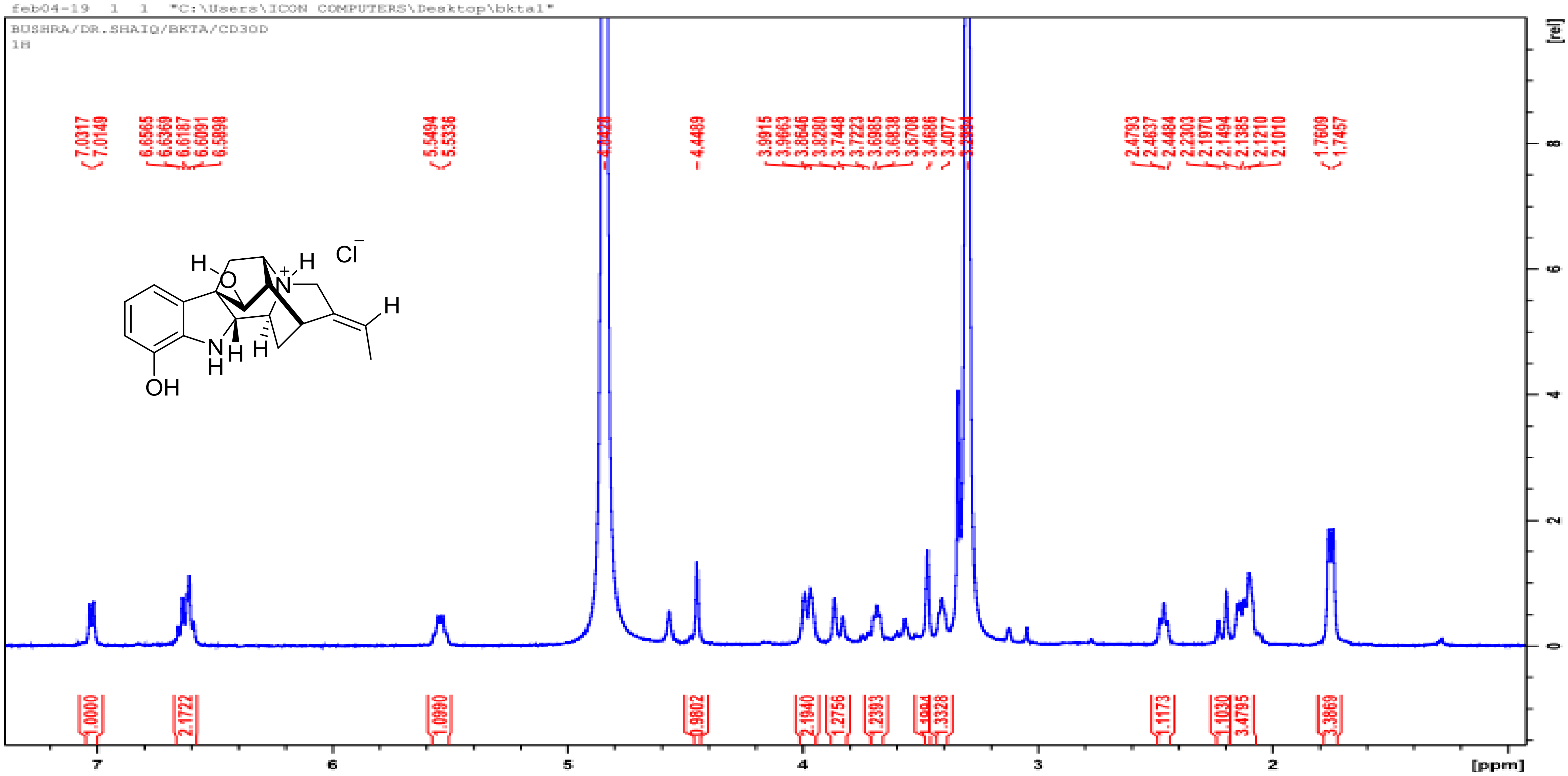

Figure S1: ^{1}H-NMR (CD_3OD, 400 MHz) Spectrum of Yaundentine hydrochloride (**1**)

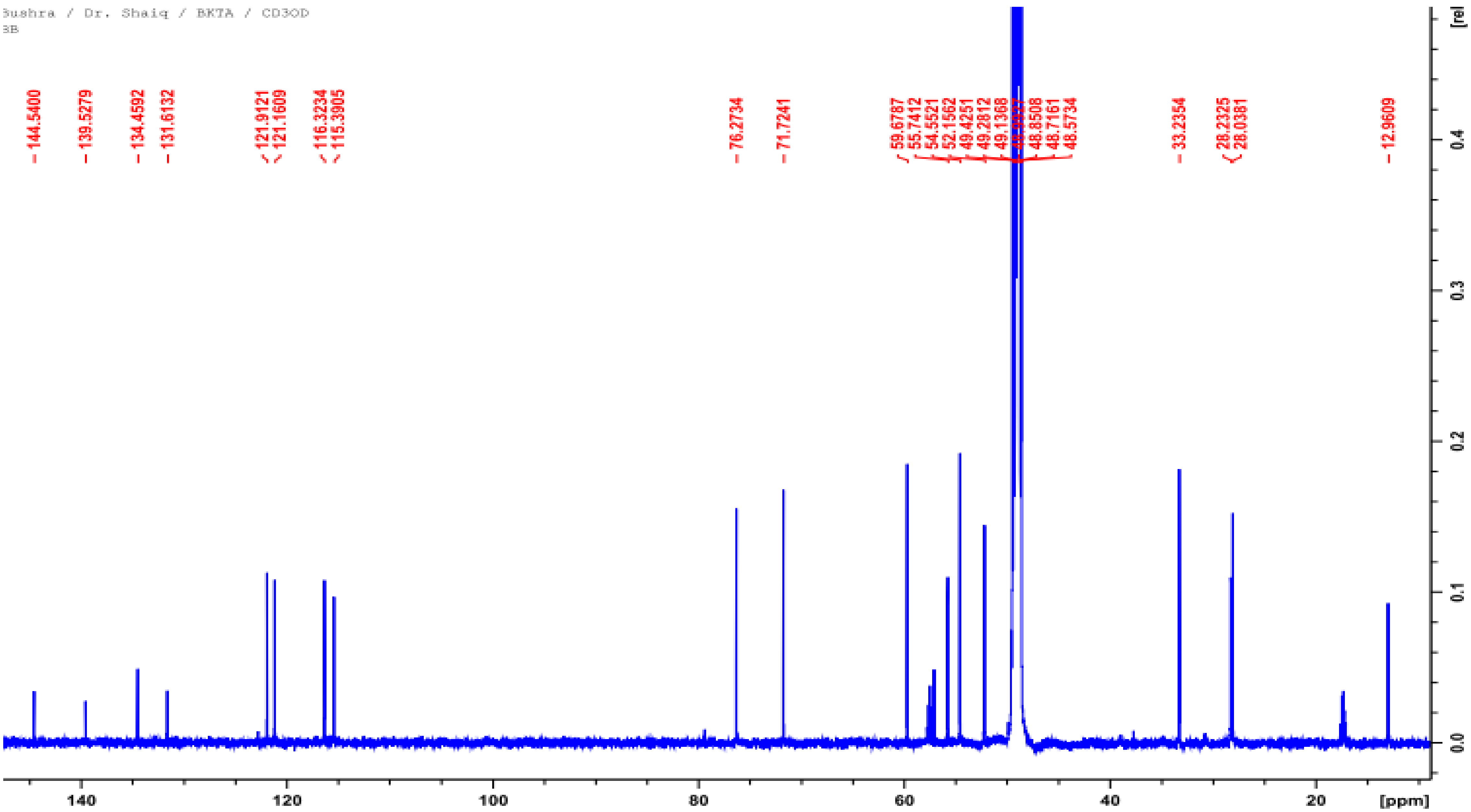

Figure S2: [13] C-NMR (CD_3OD, 100 MHz) Spectrum of Yaundentine hydrochloride (**1**)

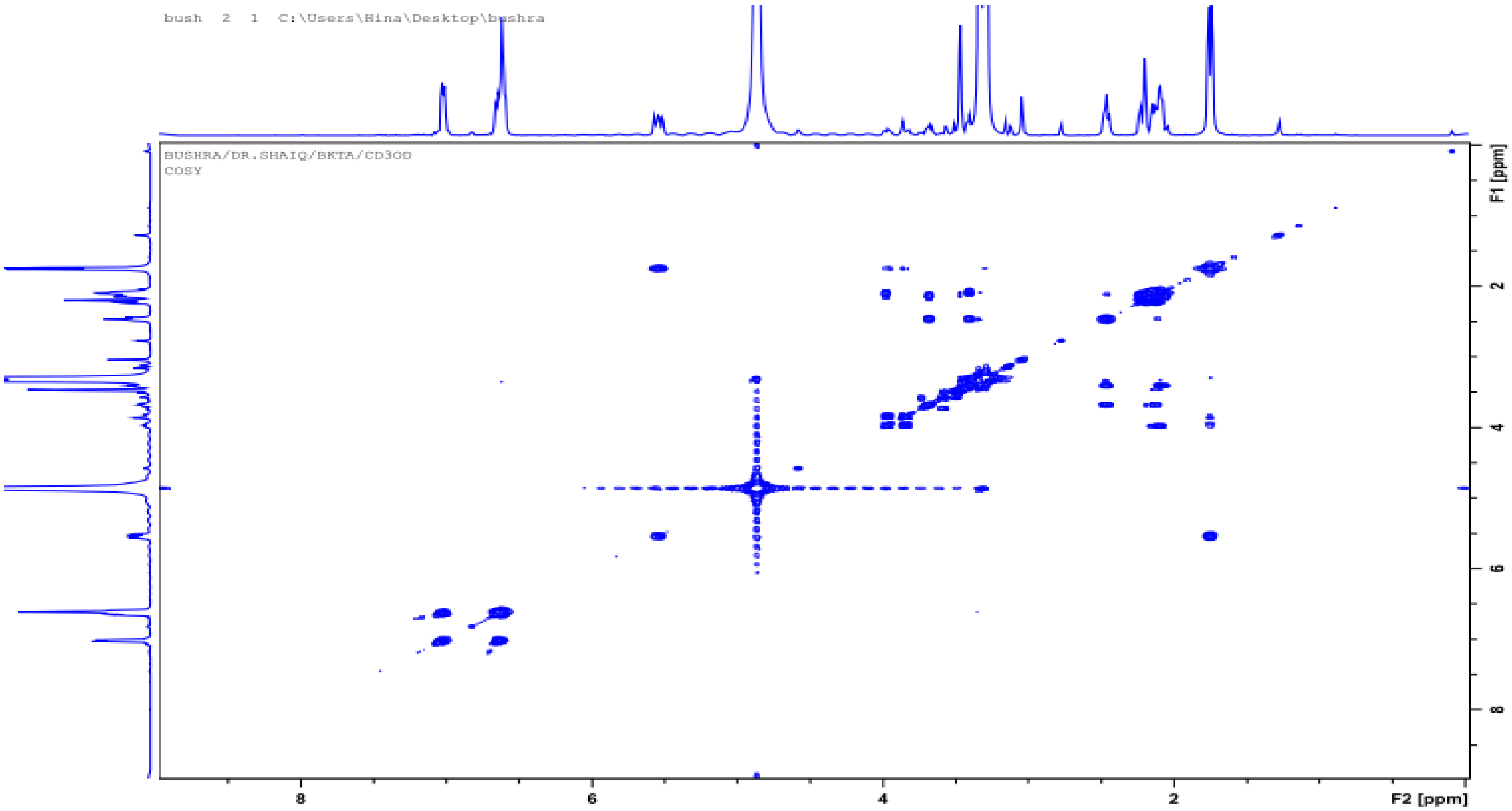

Figure S3: COSY-45° Correlations of Yaundentine hydrochloride (**1**)

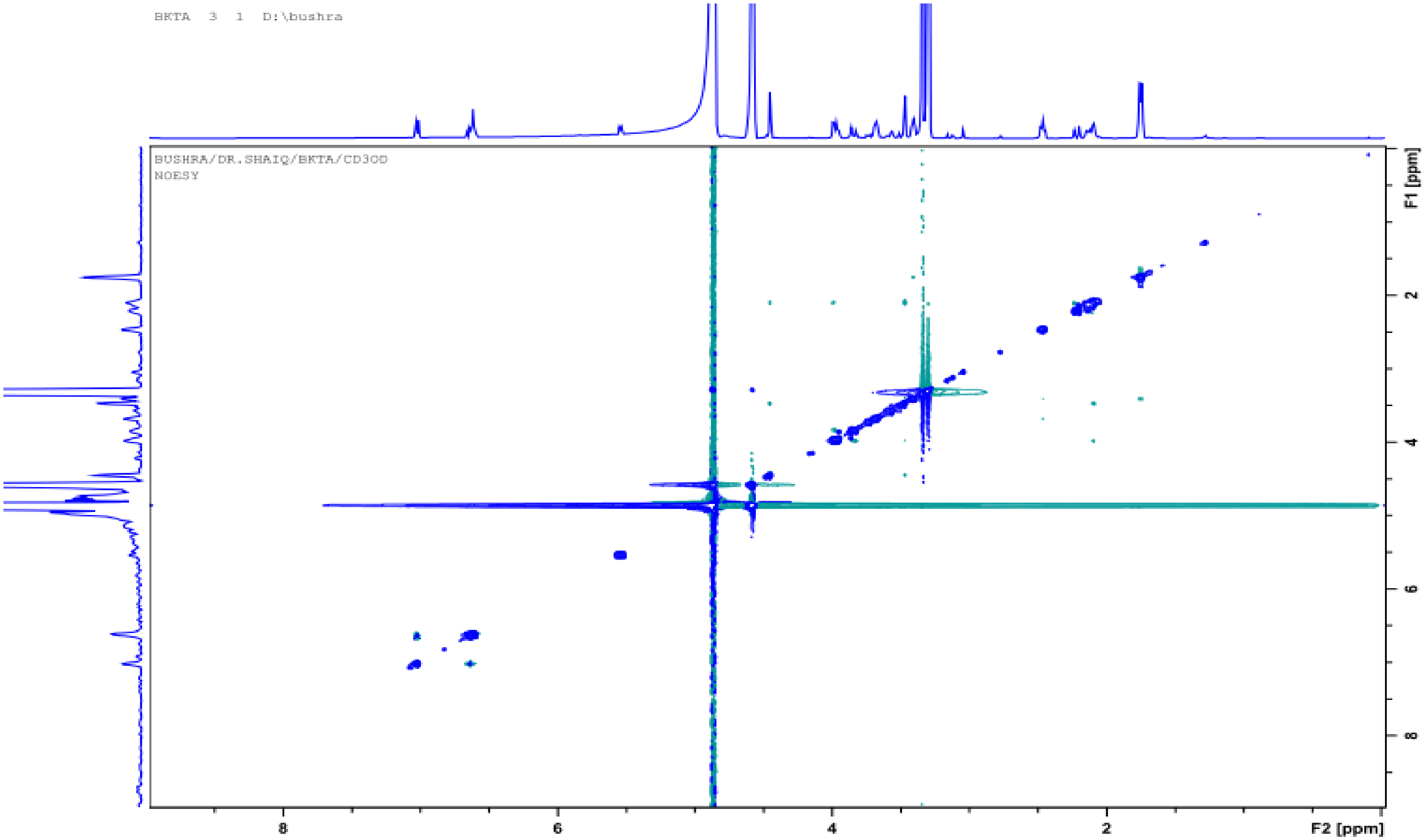

Figure S4: NOSEY Correlations of Yaundentine hydrochloride (**1**)

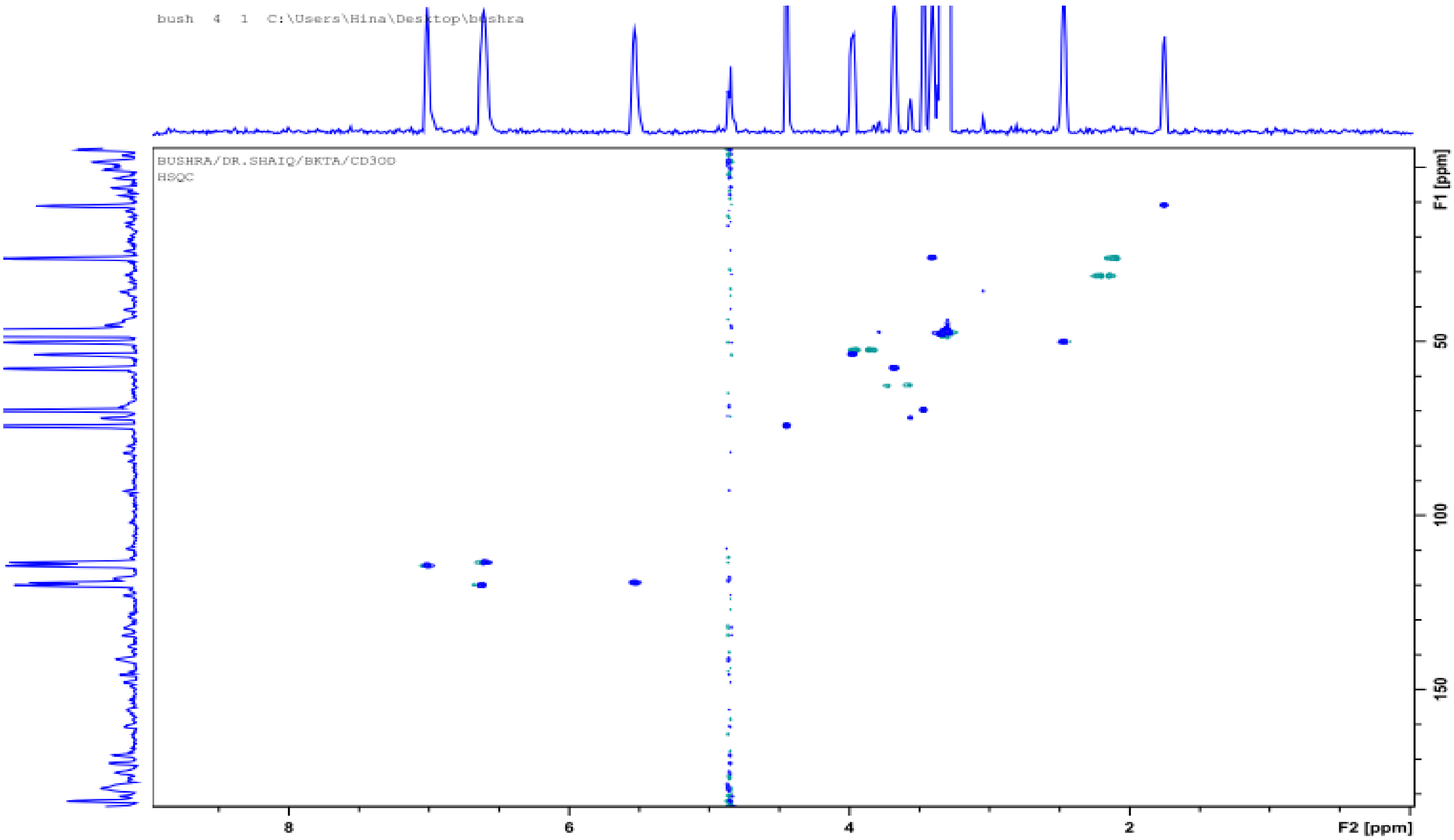

Figure S5: HSQC Correlations of Yaundentine hydrochloride (**1**)

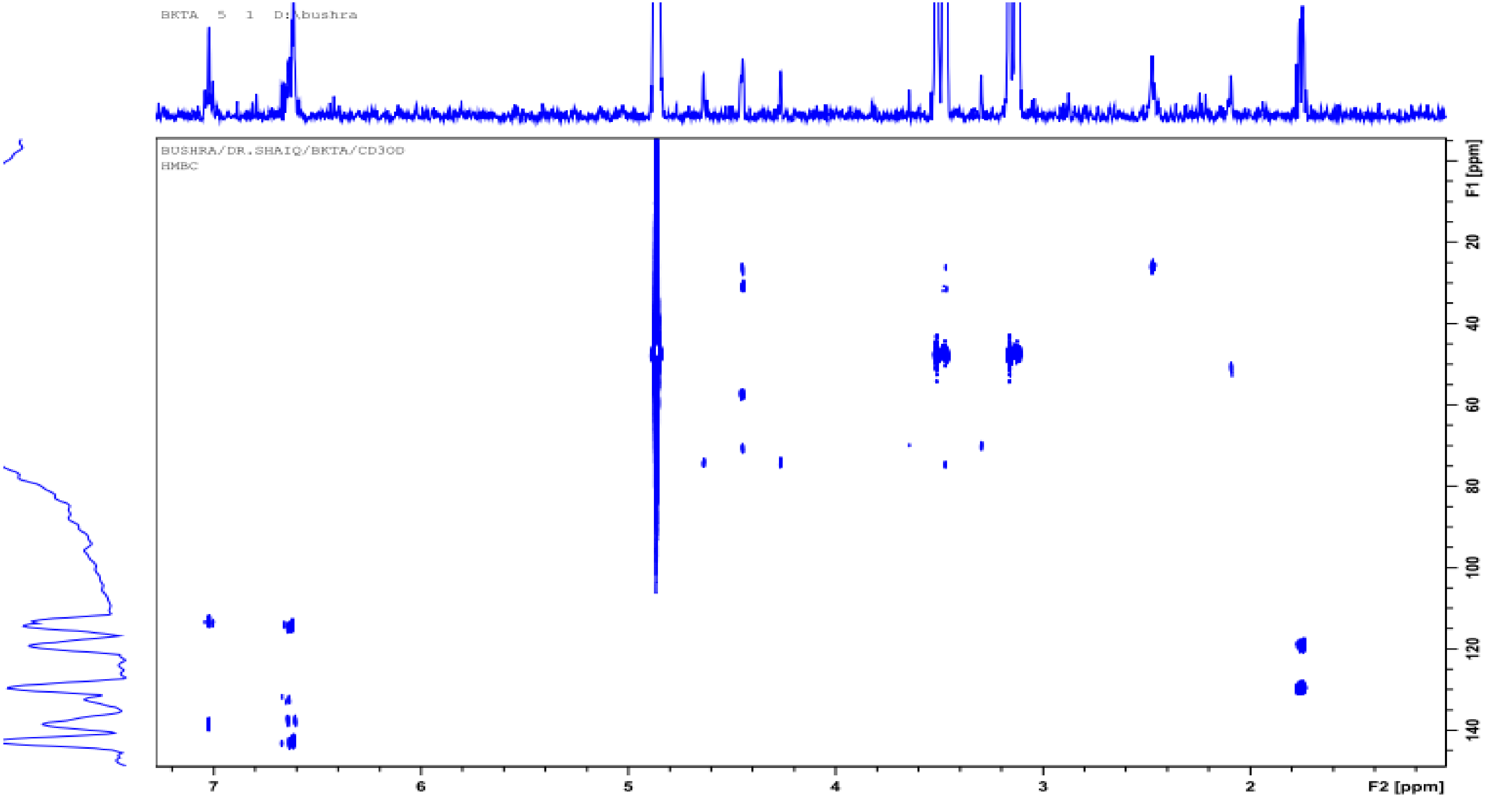

Figure S6: HMBC Correlations of Yaundentine hydrochloride (**1**)

ICCBS
9/7/2017

File: BK-I-64a
Sample: BUSHRA /DR. SHAIQ
Instrument: JEOL MSRoute
Inlet: Direct Probe

Date Run: 09-07-2017 (Time Run: 11:51:29)

Ionization mode: EI+

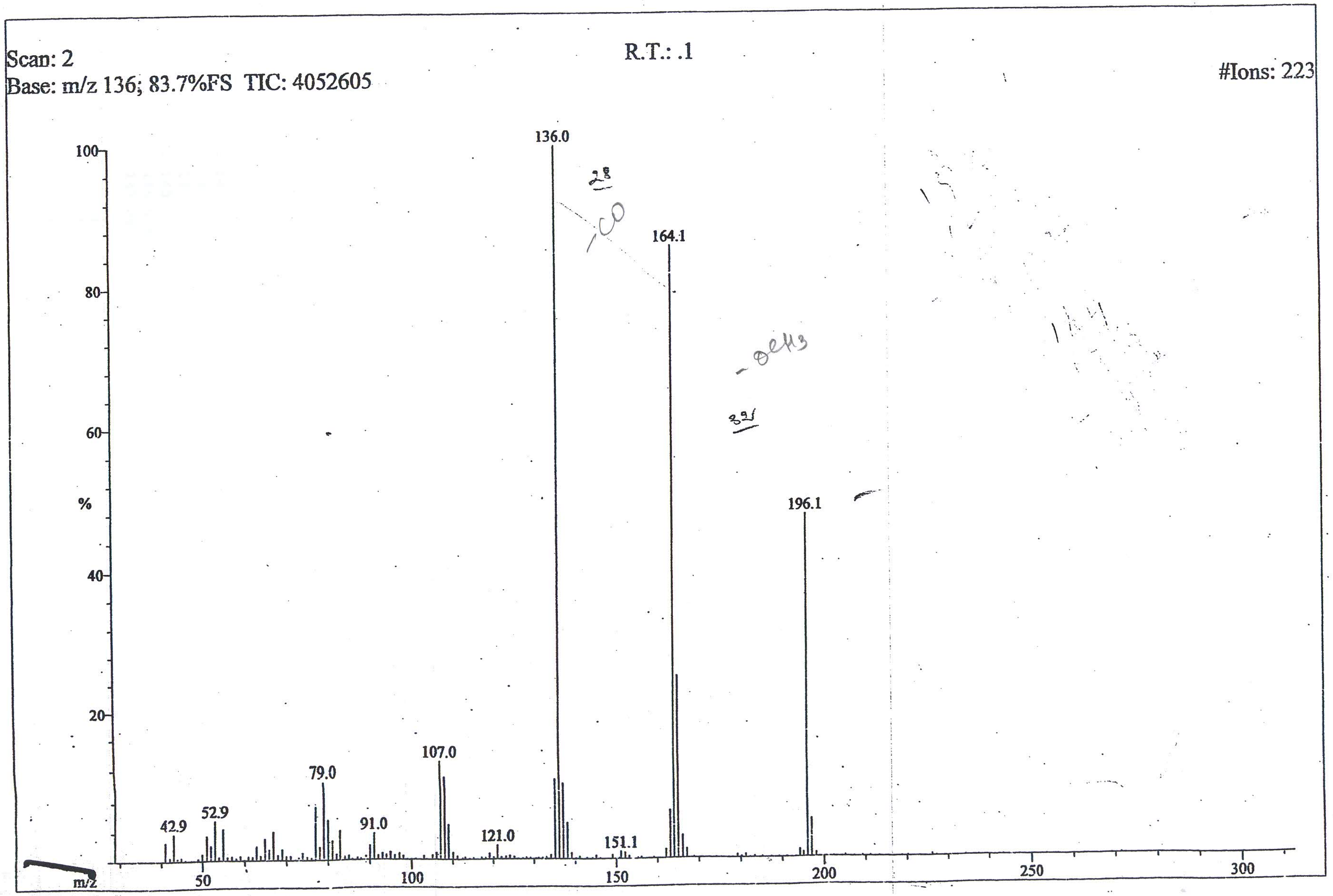

Figure S7: EIMS Spectrum of of Methyl -orcinol carboxylate

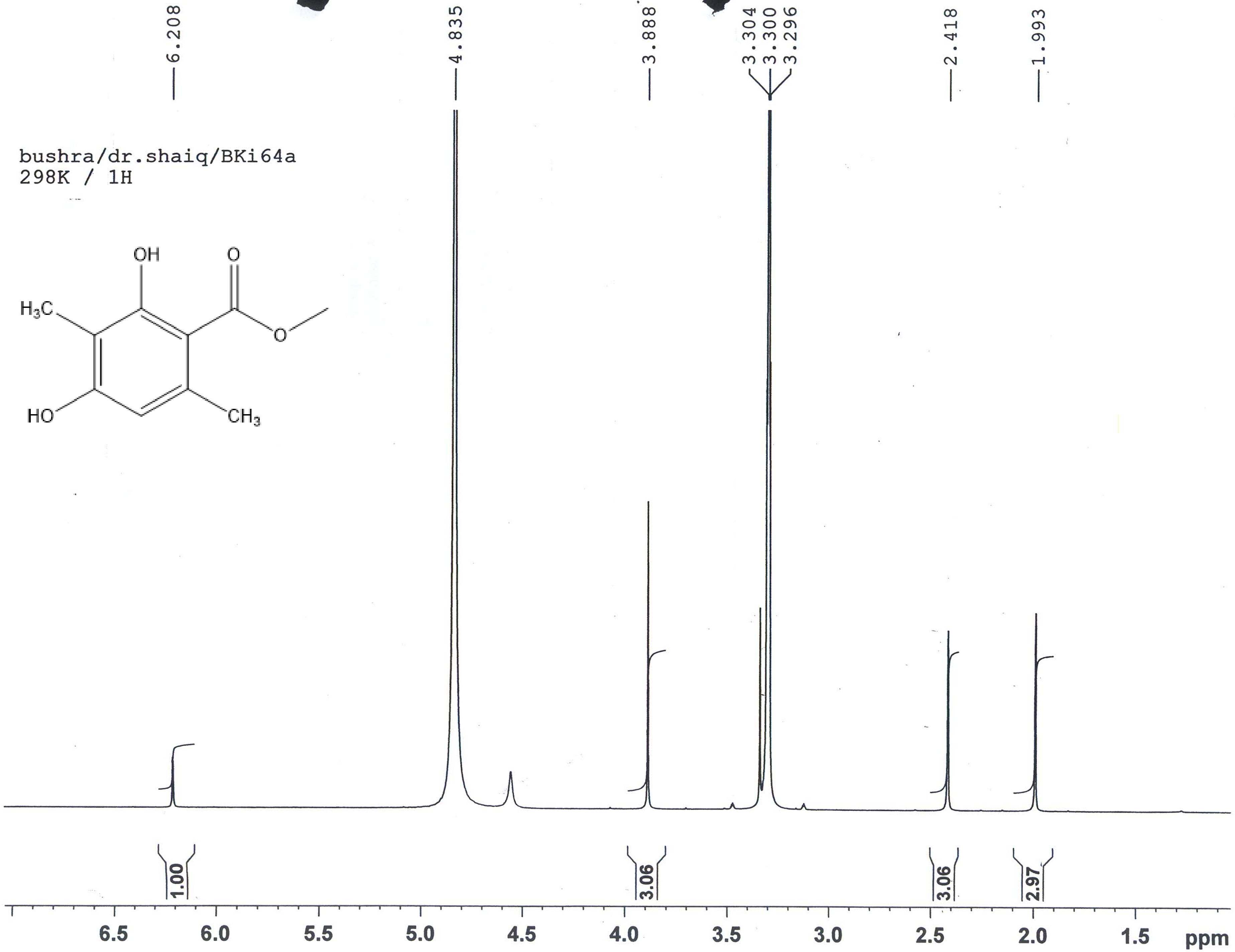

Figure S8: ^{1}H-NMR (CD3OD, 400 MHz) Spectrum of of Methyl -orcinol carboxylate

ICCBS
1/9/2018 3:41:31 PM

Page 1

File: BKE6

Date Run: 01-09-2018 (Time Run: 14:56:29)

Sample: BUSHRA / DR. SHAIQ

Instrument: JEOL MSRoute

Run By: HEJ-ICCBS

Inlet: Direct Probe

Ionization mode: EI+

Scan: 49

R.T.: 4.23

Base: m/z 189; 85.2%FS TIC: 16620603

#Ions: 689

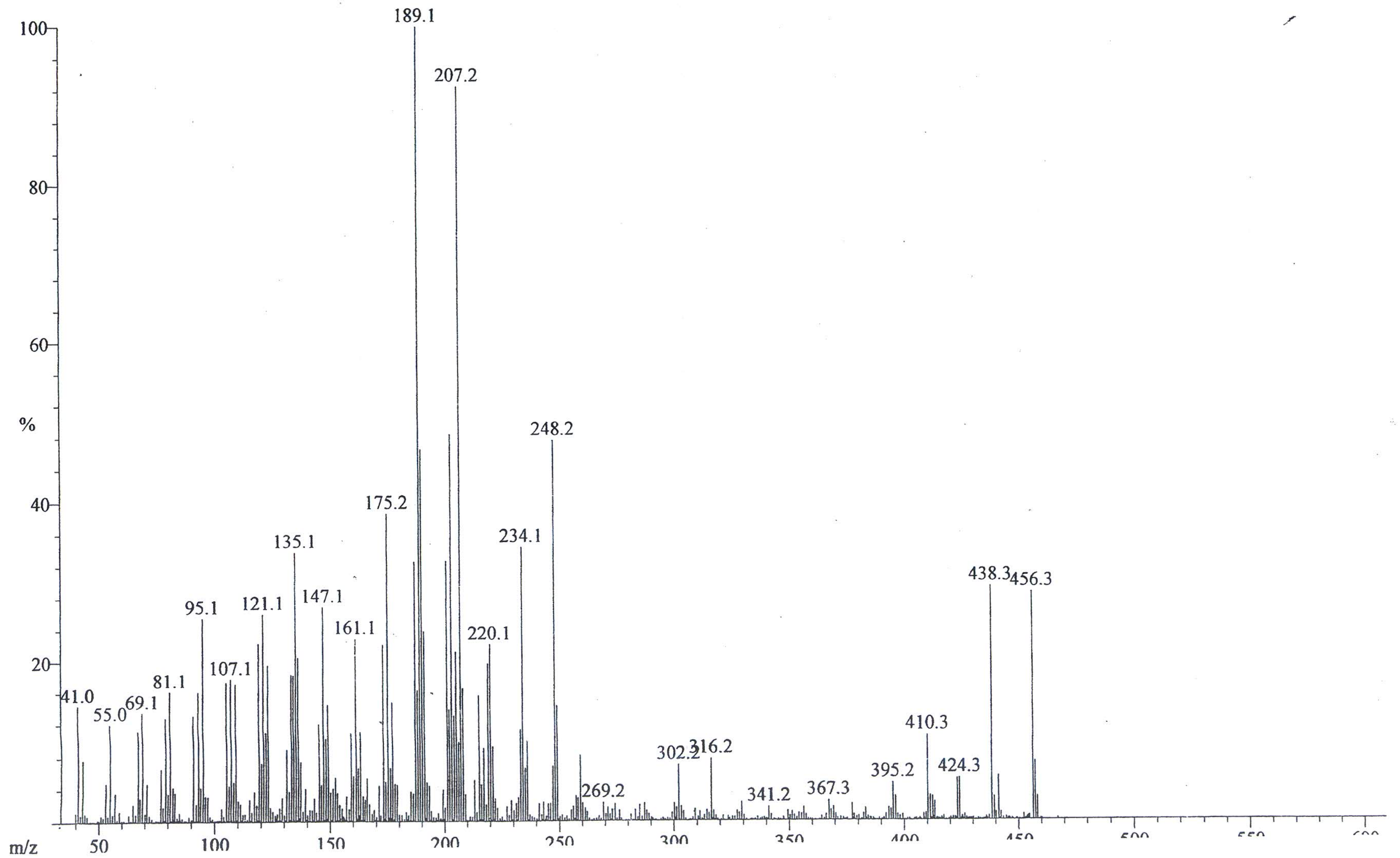

Figure S9: EIMS Spectrum of Betulinic Acid

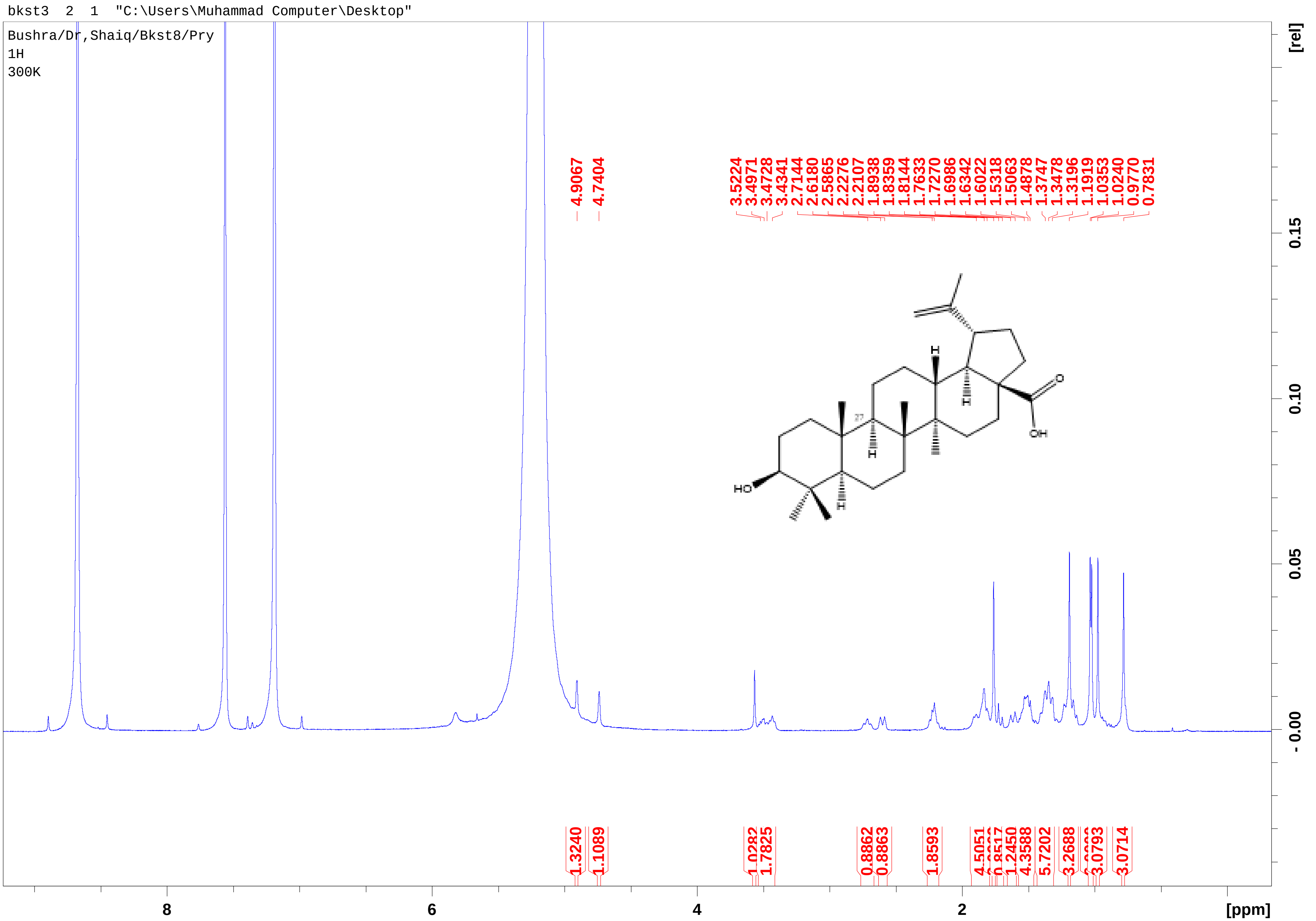

Figure S10: ¹H-NMR (C5D5N, 400 MHz) Spectrum of Betulinic Acid

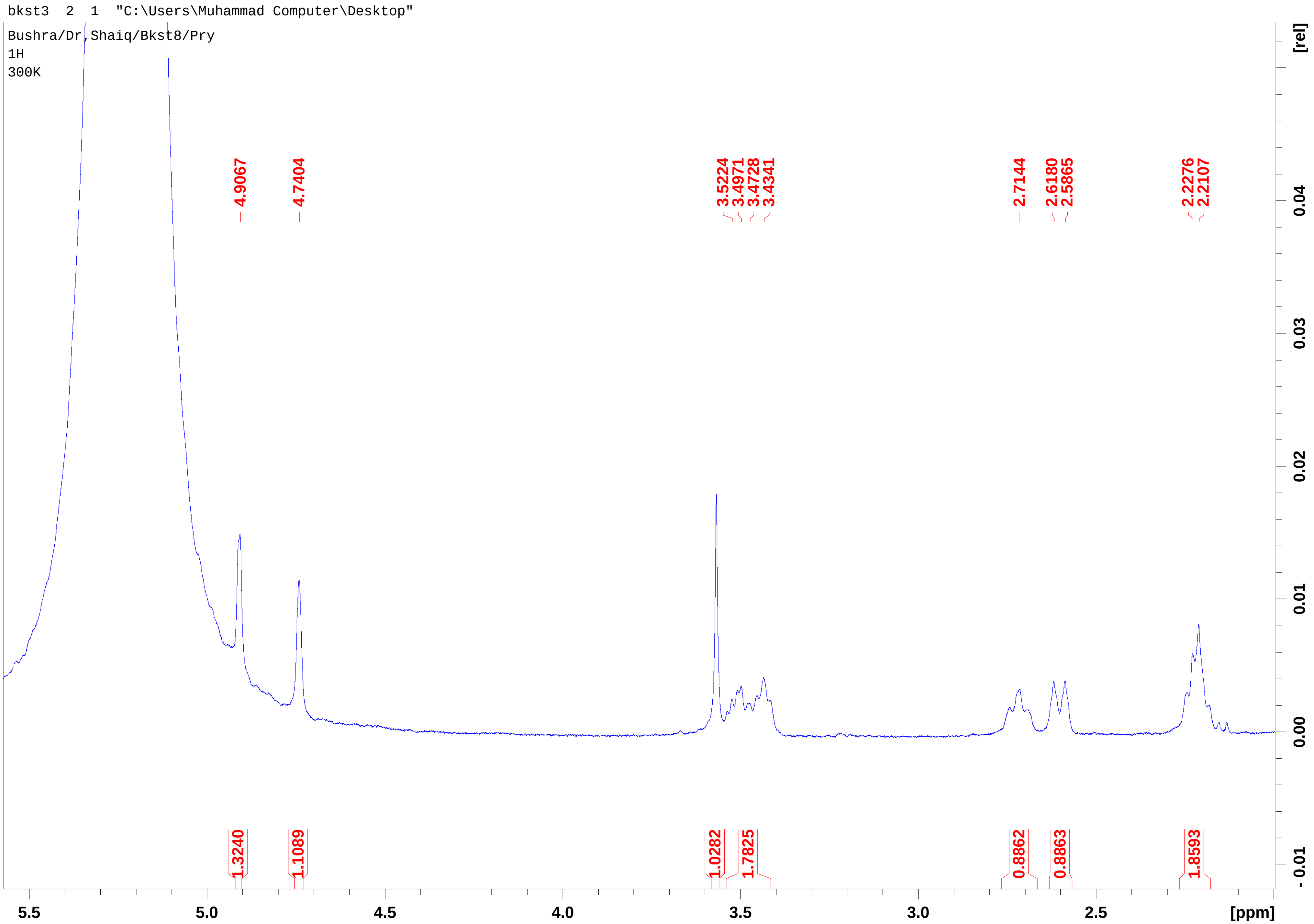

Figure S11: ^{1}H-NMR (C5D5N, 400 MHz) Spectrum of Betulinic Acid

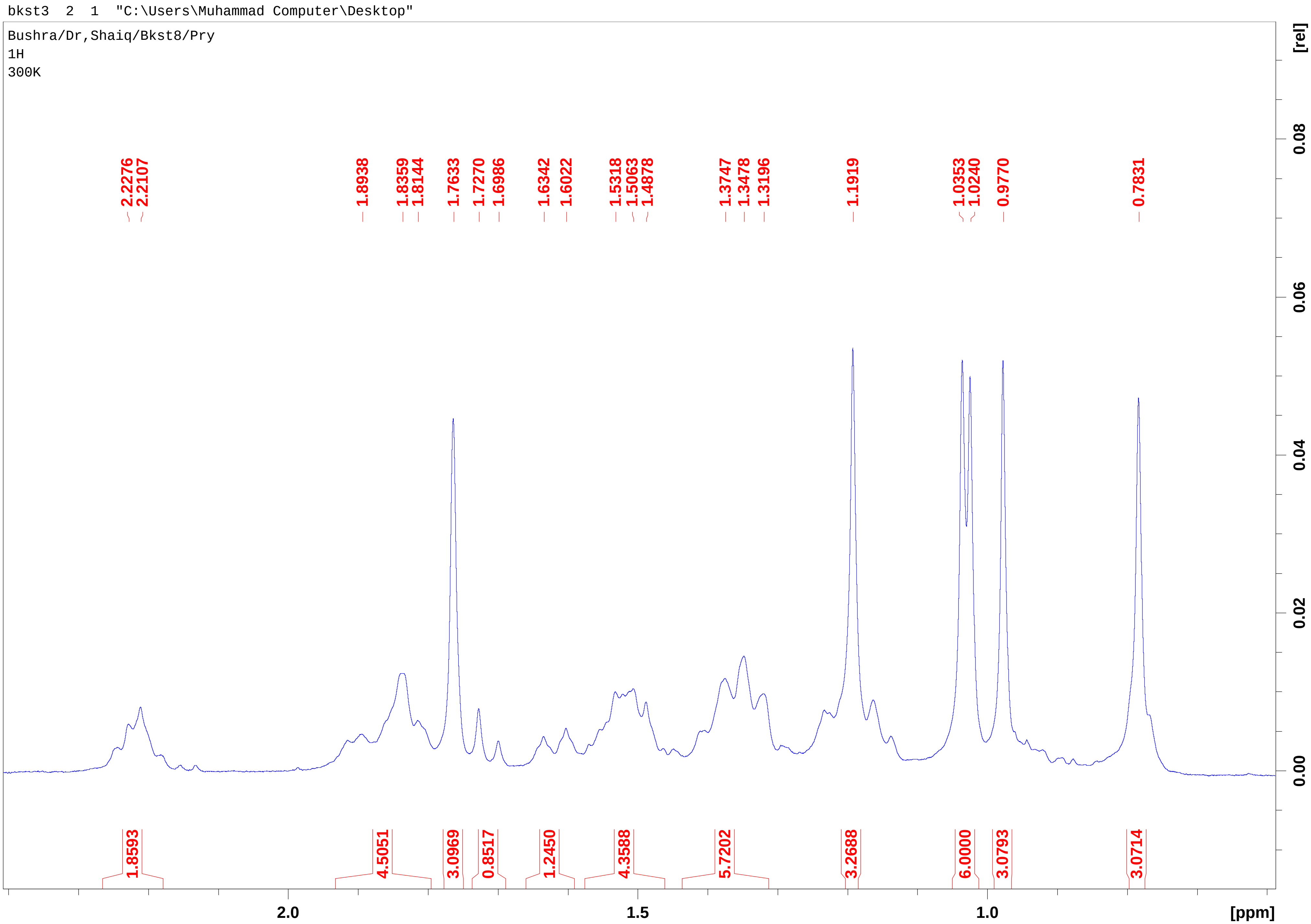

Figure S12: ¹H-NMR (C5D5N, 400 MHz) Spectrum of Betulinic Acid

feb13-18 7 1 "D:\No.2 [NTFS]\Root Dir"

BUSHRA/DR,SHAIQ/BK-be/
ICCBS,U.O.K/1H

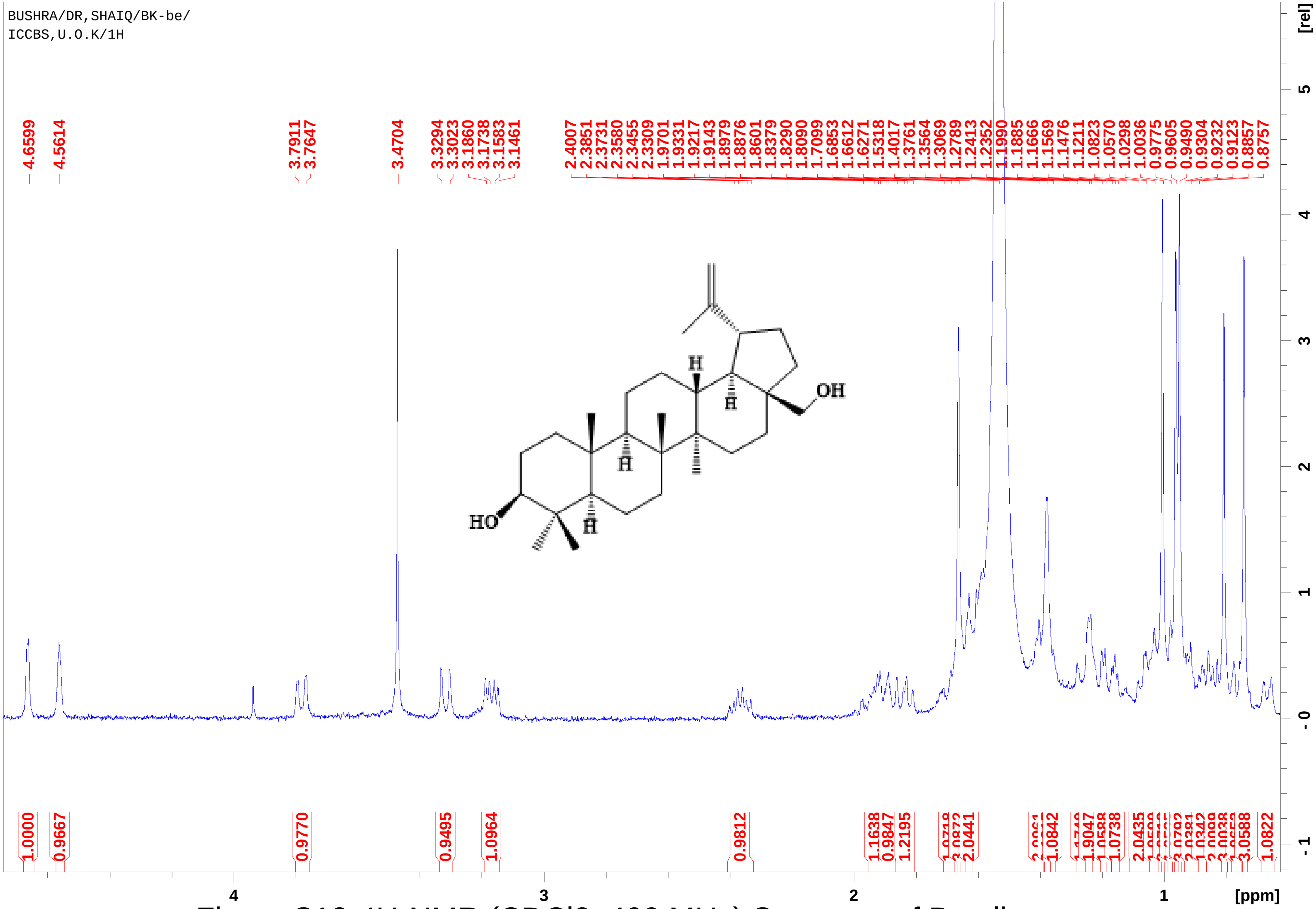

Figure S13: ¹H-NMR ($CDCl_3$, 400 MHz) Spectrum of Betulin

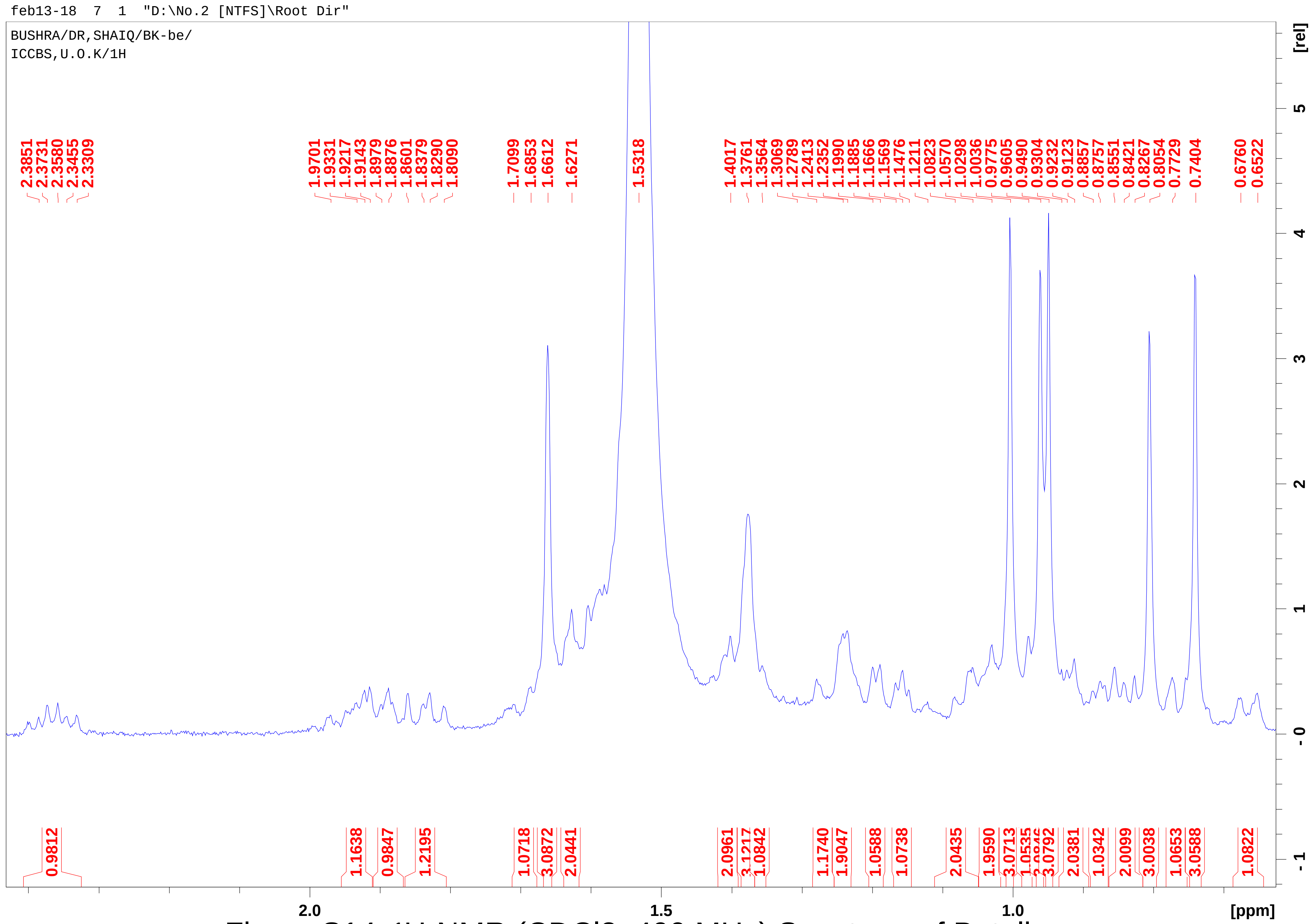

Figure S14: ^{1}H-NMR (CDCl3, 400 MHz) Spectrum of Betulin

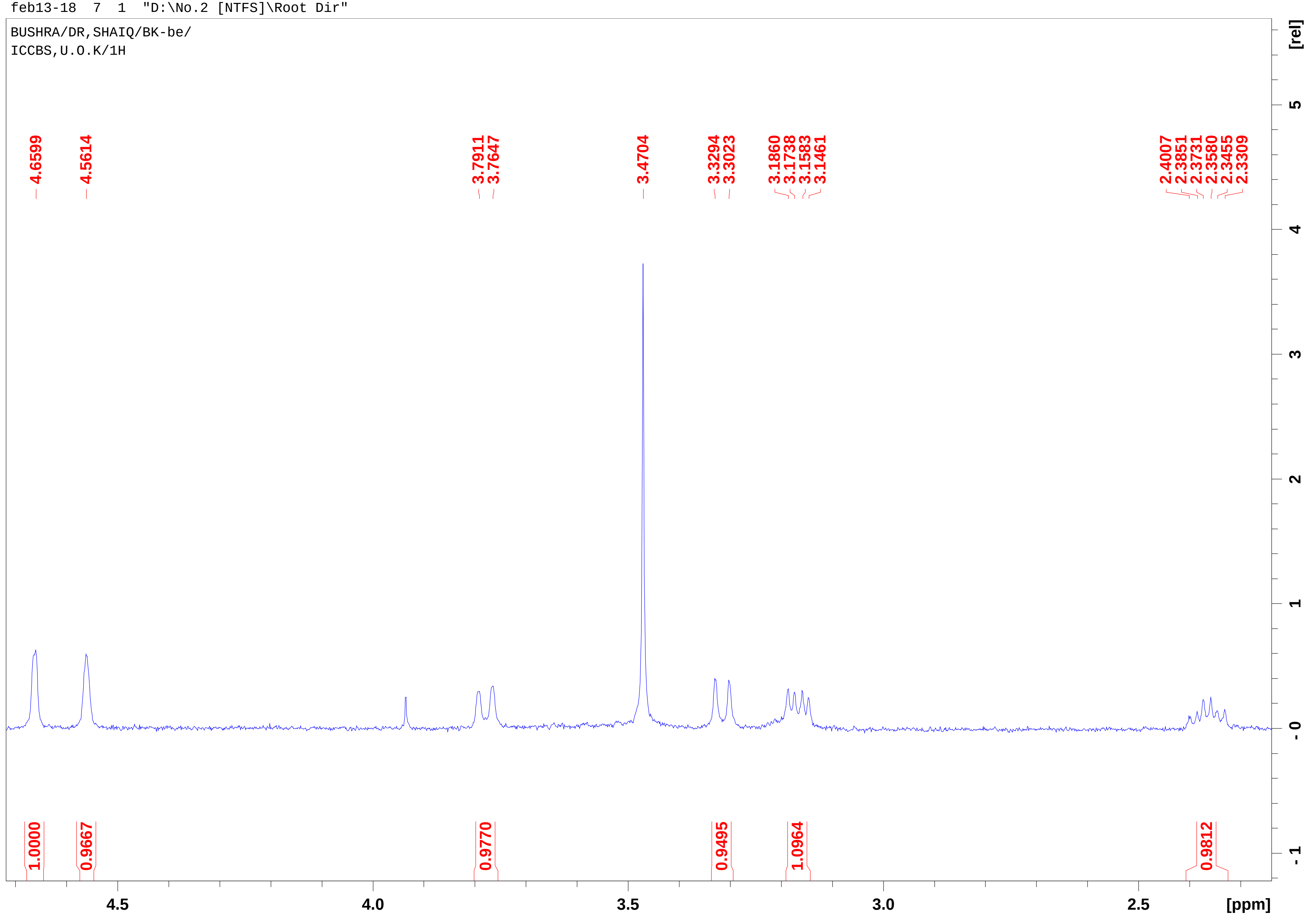

Figure S15: ¹H-NMR (CDCl3, 400 MHz) Spectrum of Betulin

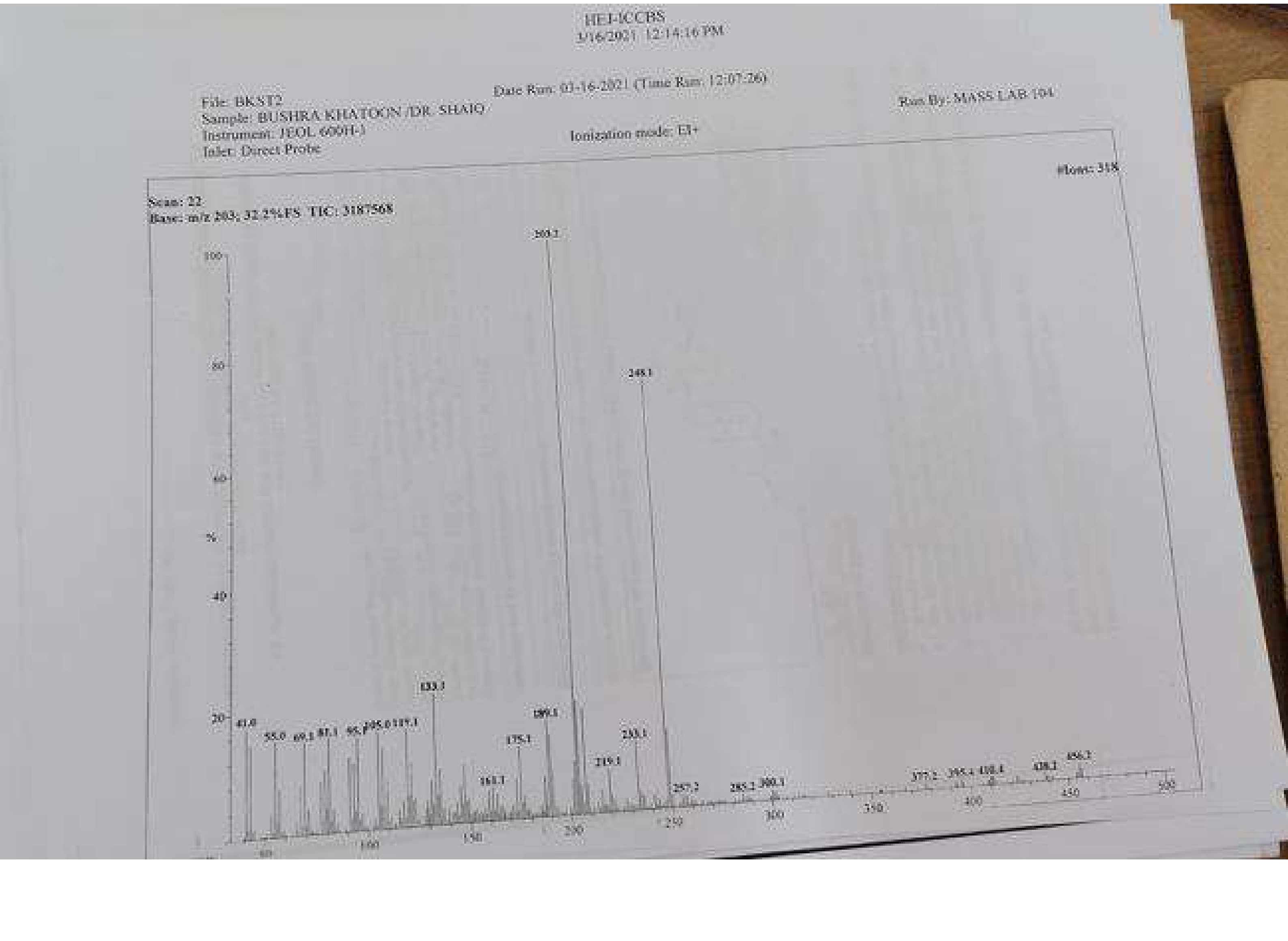

Figure S16: EIMS Spectrum of Oleanolic Acid

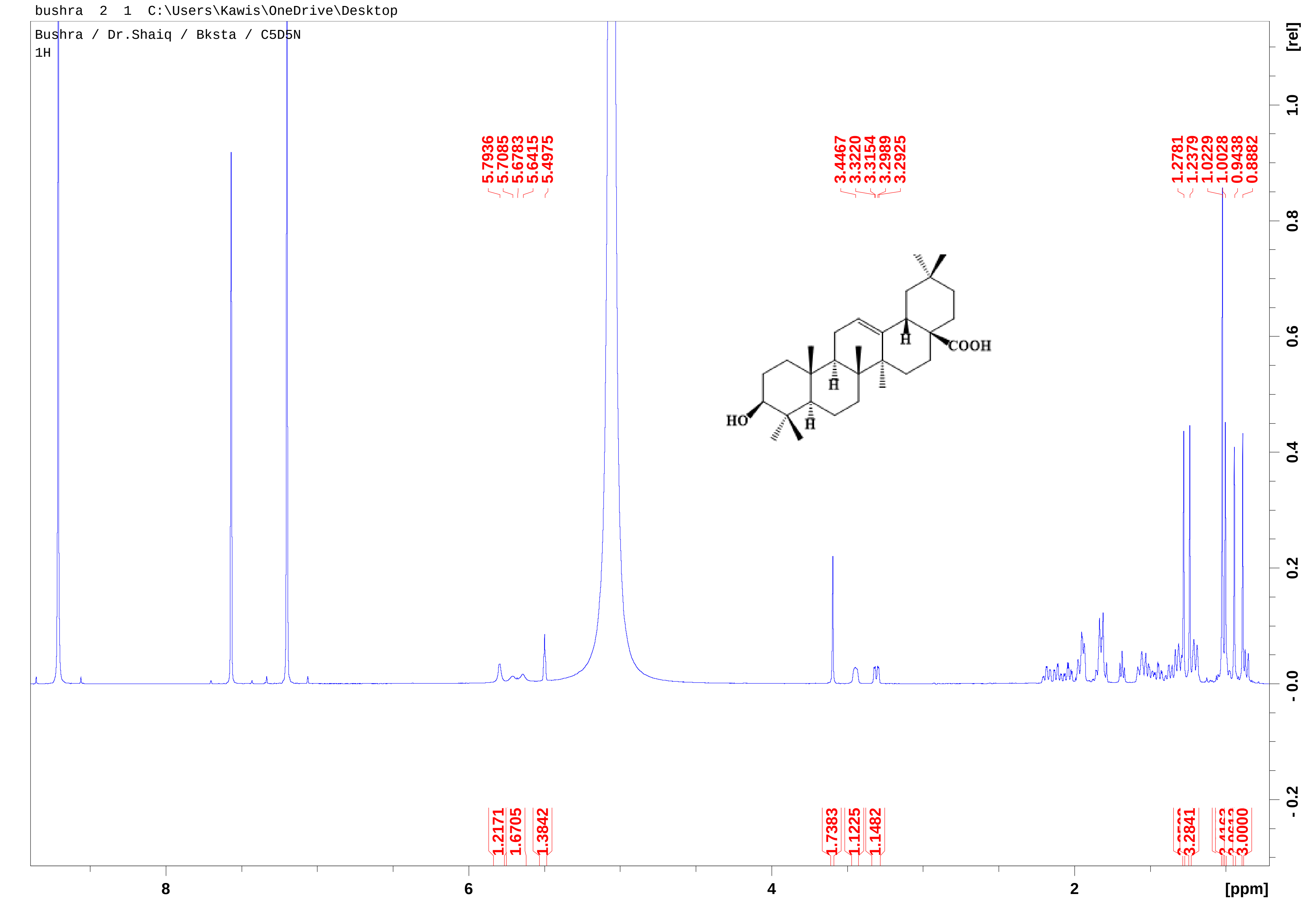

Figure S17: ¹H-NMR (C5D5N, 400 MHz) Spectrum of Oleanolic Acid

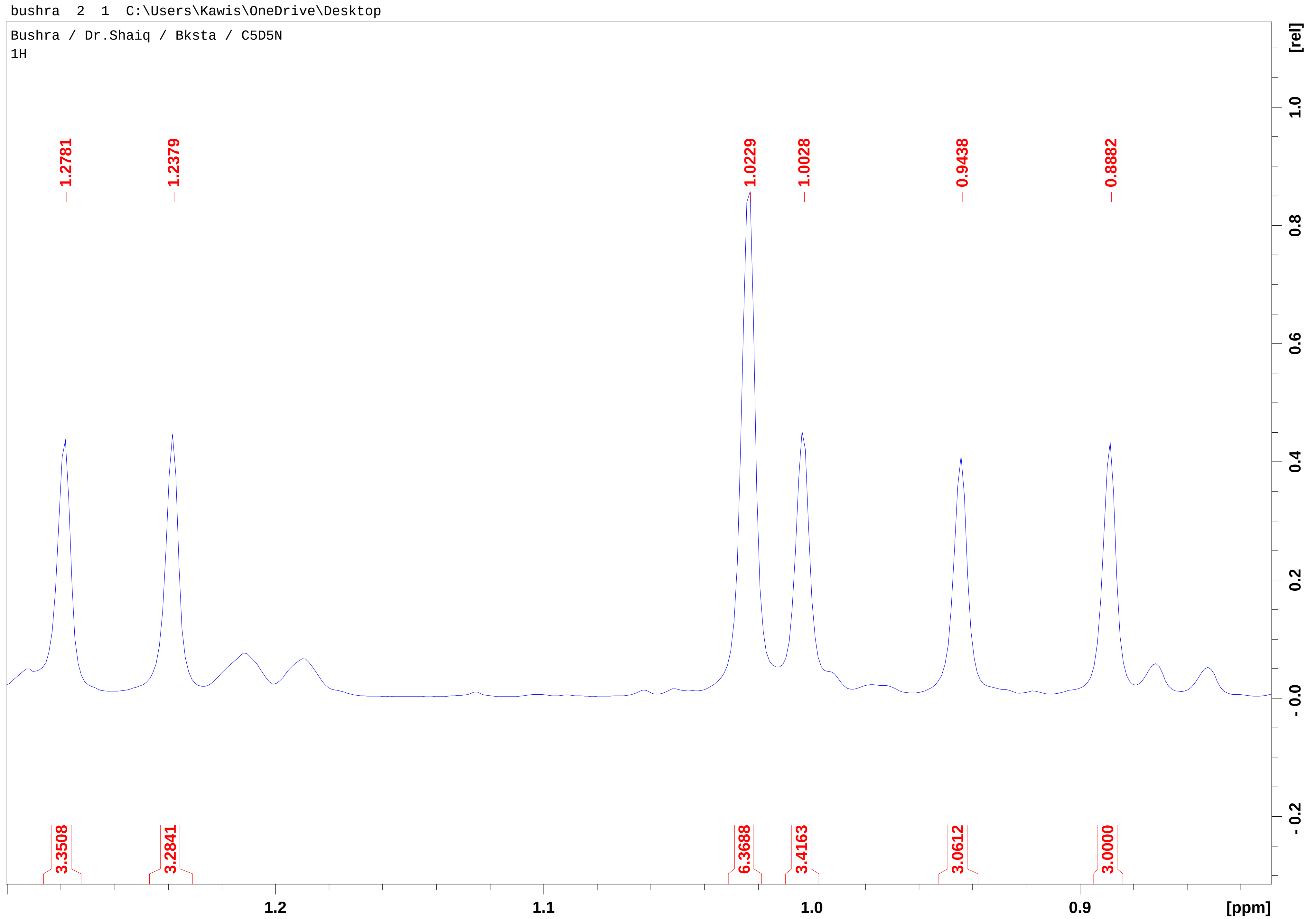

Figure S18: ¹H-NMR (C5D5N, 400 MHz) Spectrum of Oleanolic Acid

HEJ-ICCBS
11/10/2020 2:28:39 PM

File: BKST5 Date Run: 11-10-2020 (Time Run: 14:19:40)
Sample: BUSHRA KHATOON /DR. SHAIQ ALI
Instrument: JEOL 600H-1 Run By: MASS LAB-104
Inlet: Direct Probe Ionization mode: EI+

Scan: 3
Base: m/z 168; 99.9%FS TIC: 4855344 #Ions: 400

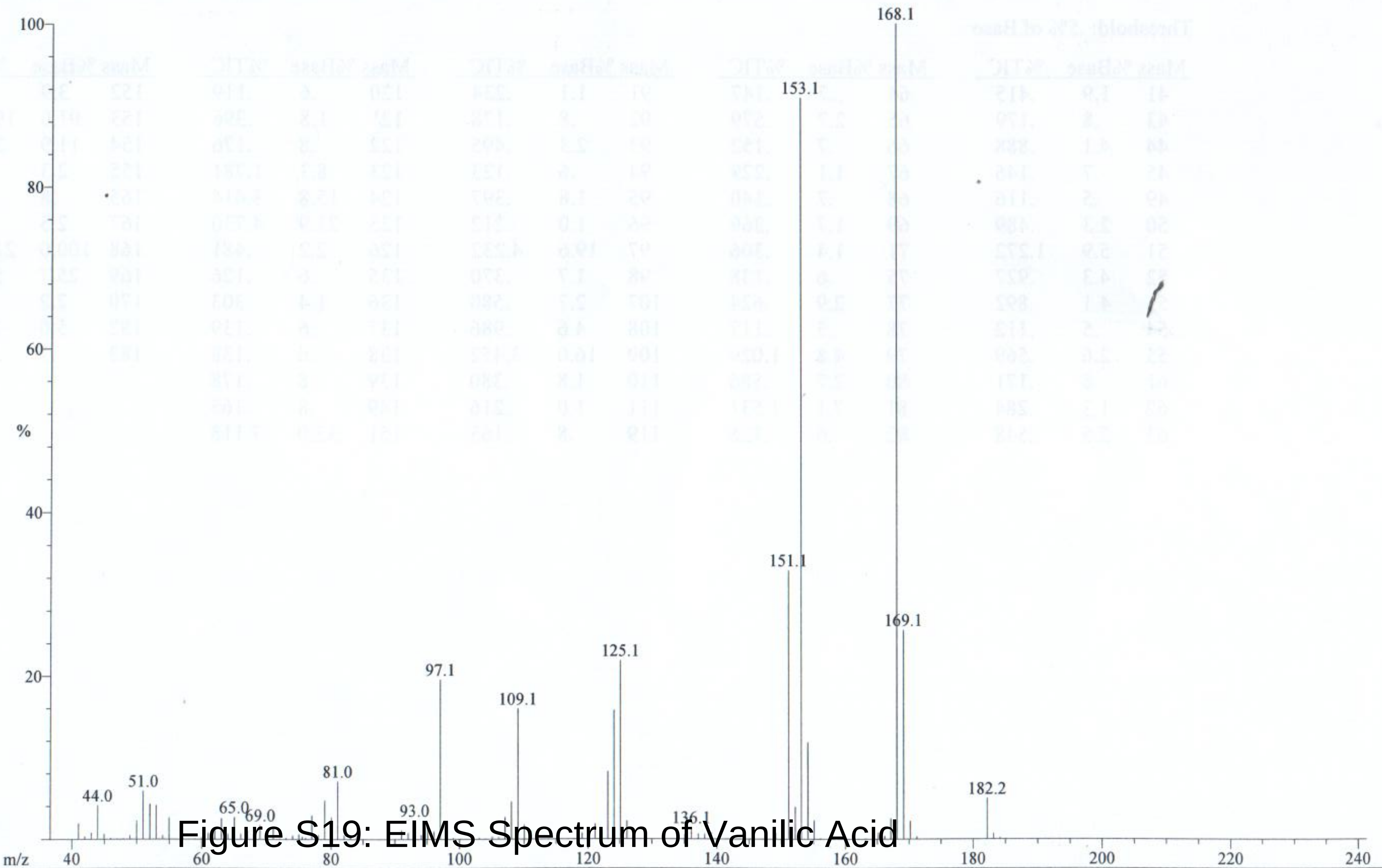

Figure S19: EIMS Spectrum of Vanilic Acid

"New folder" 8 1 "C:\Users\Muhammad Computer\Desktop"

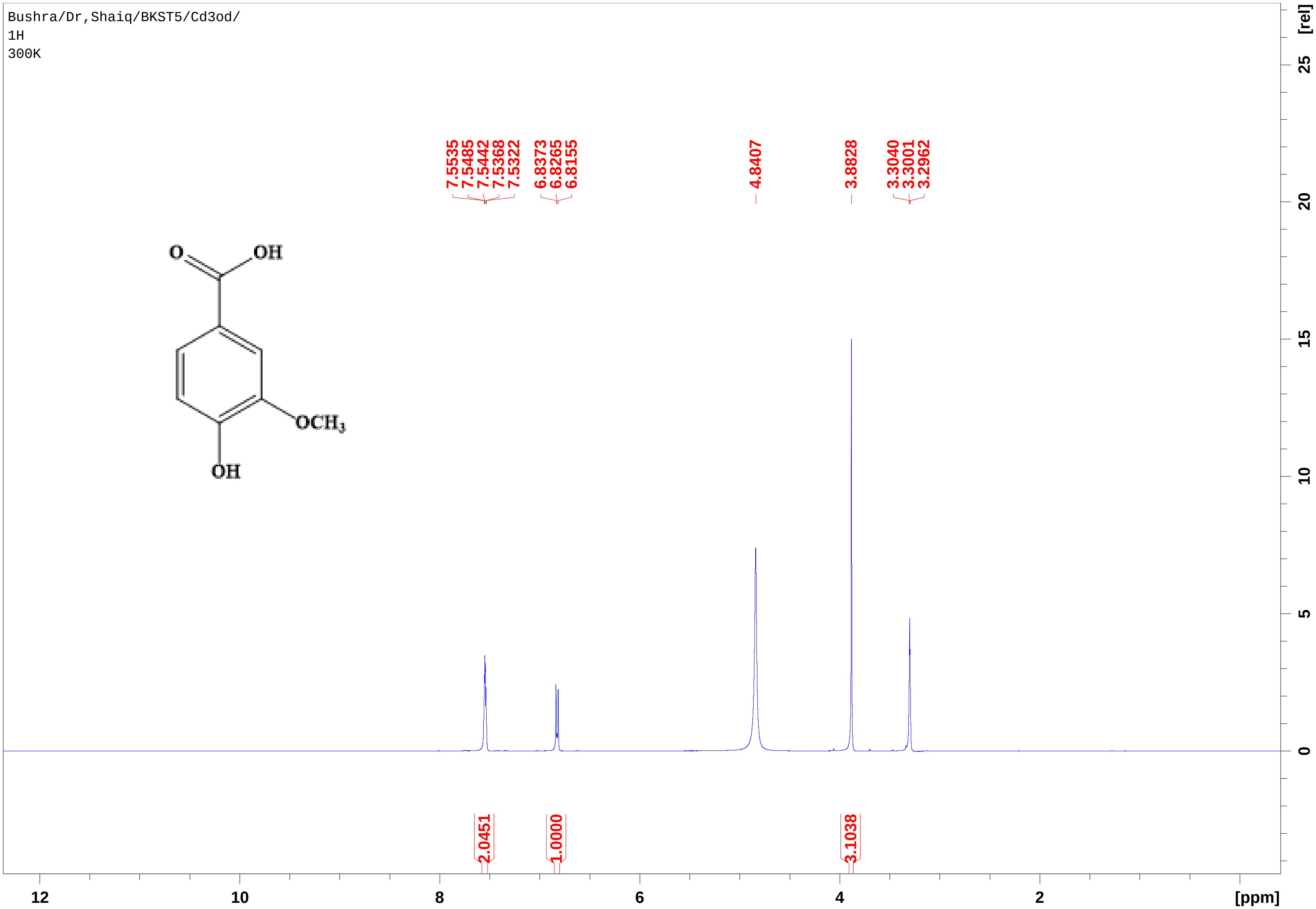

Figure S20: ¹H-NMR (CD3OD, 400 MHz) Spectrum of Vanilic Acid

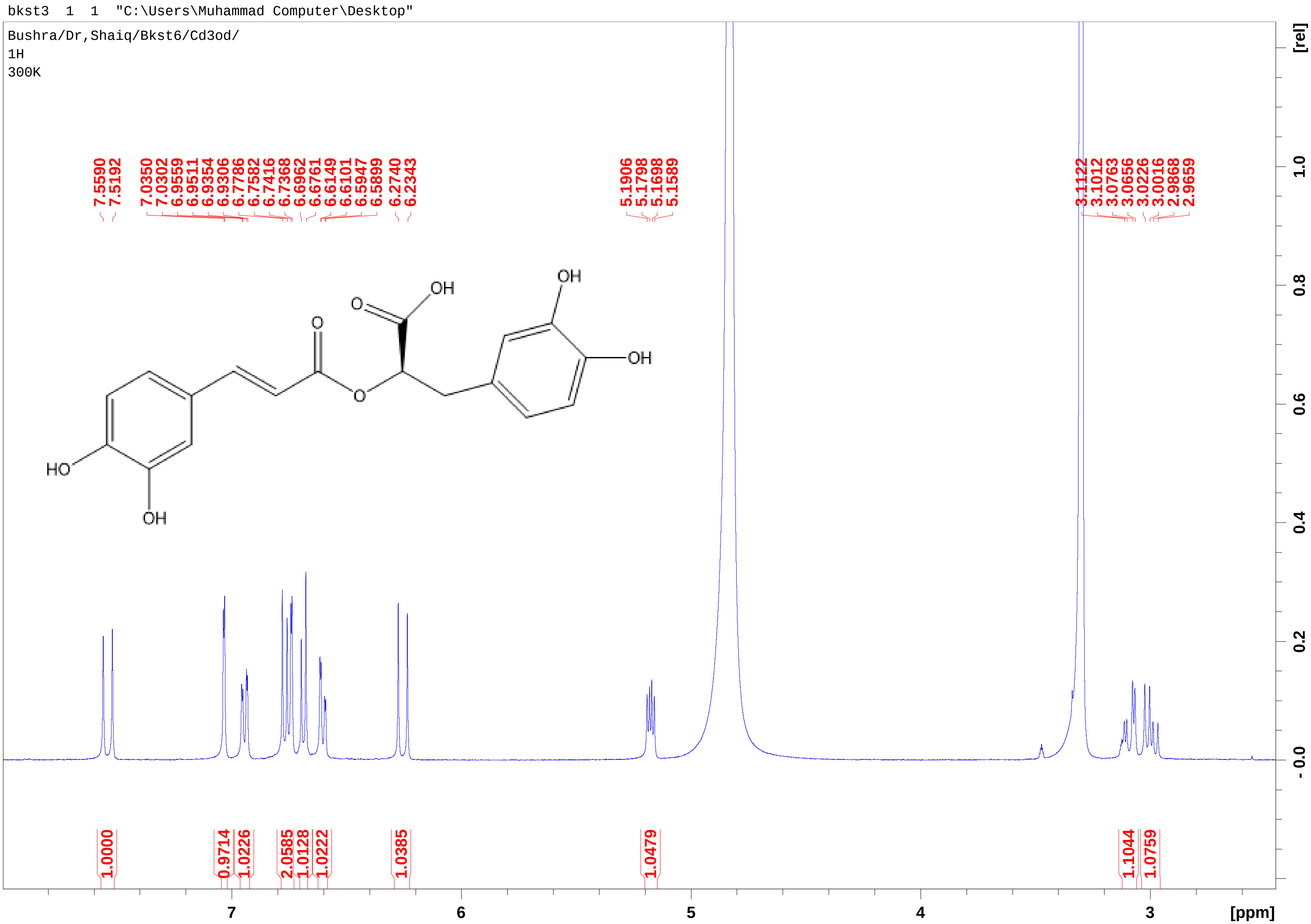

Figure S21: ¹H-NMR (CD3OD, 400 MHz) Spectrum of Rosmarinic Acid

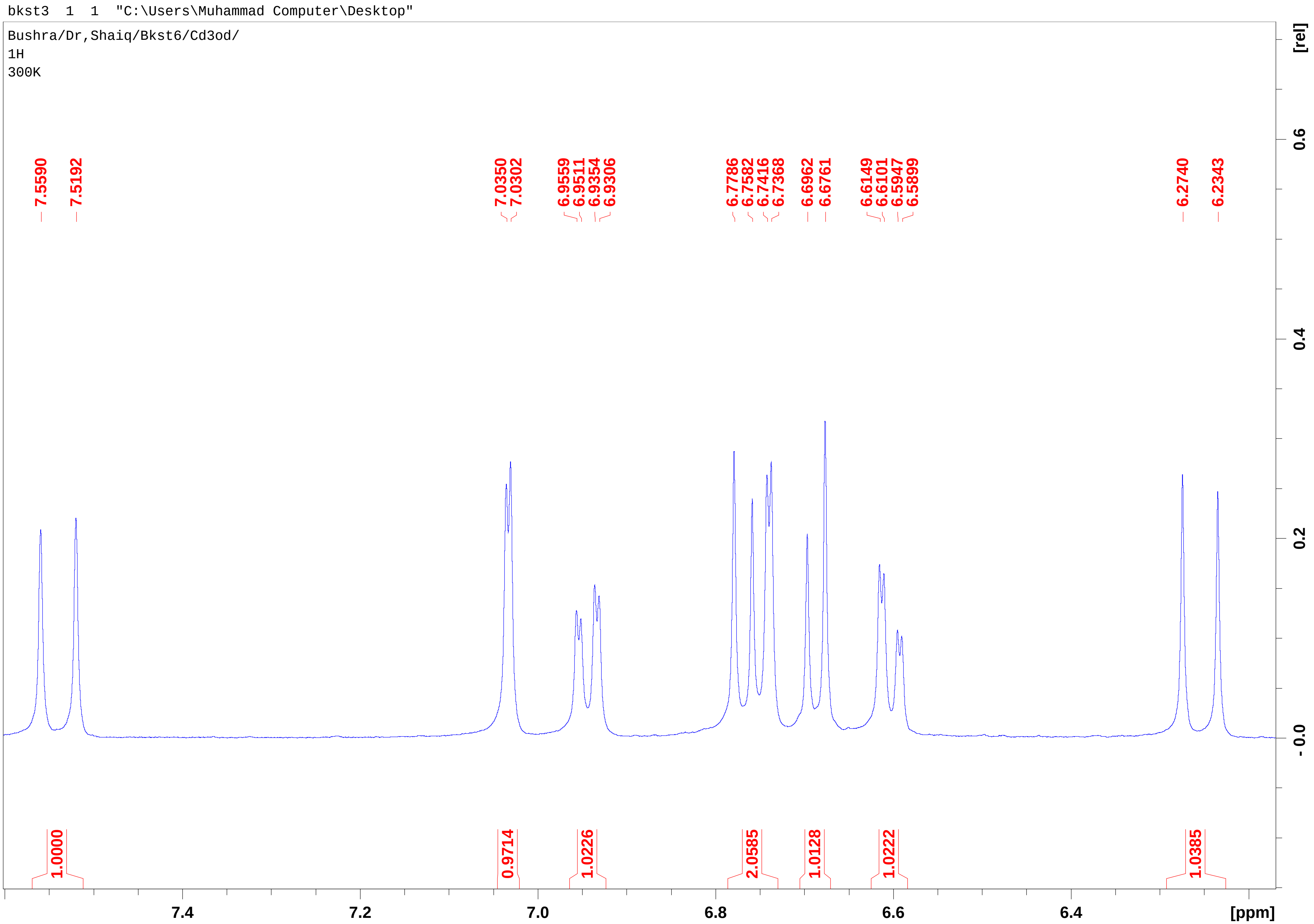

Figure S22: ^{1}H-NMR (CD3OD, 400 MHz) Spectrum of Rosmarinic Acid

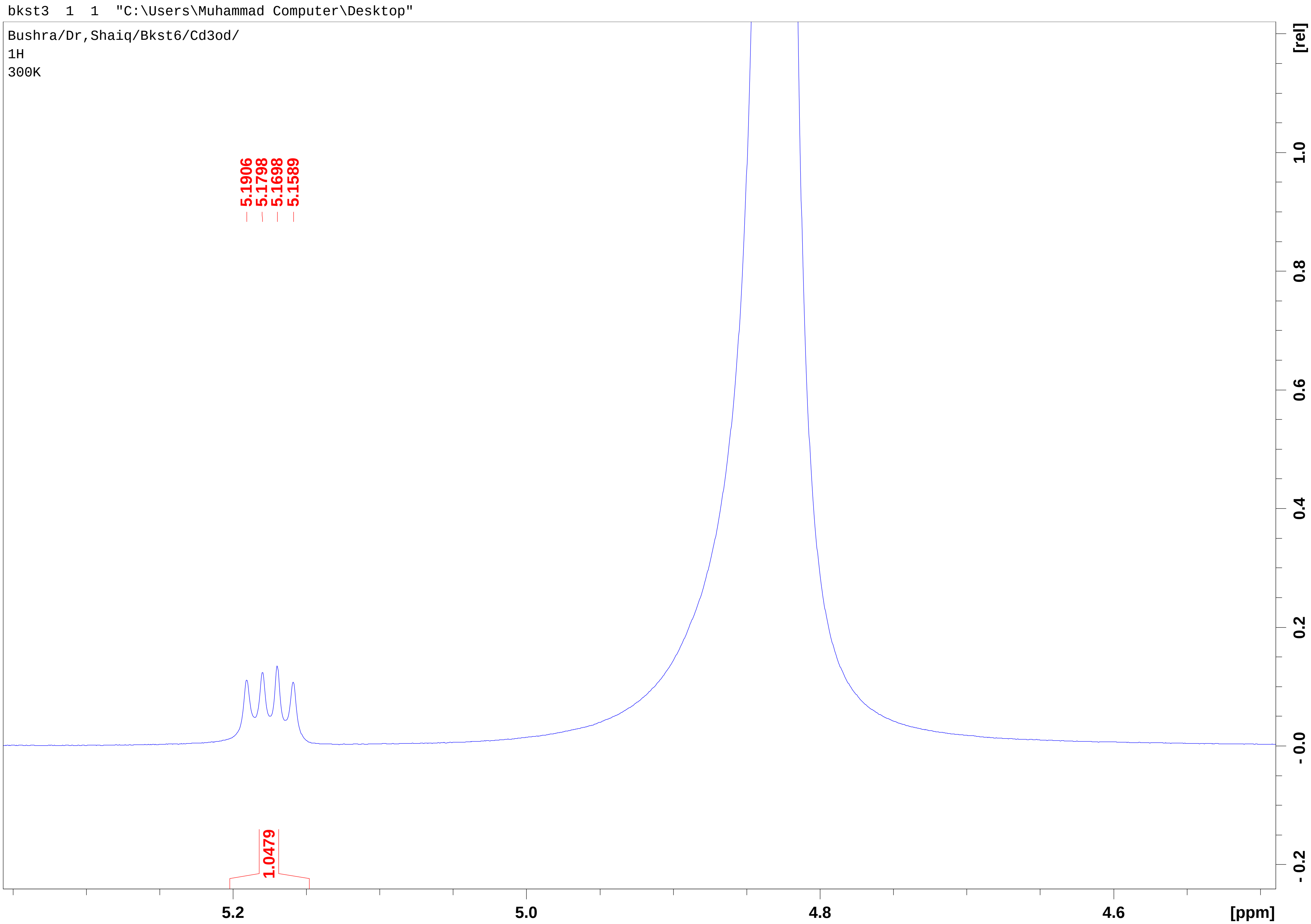

Figure S23: ^{1}H-NMR (CD3OD, 400 MHz) Spectrum of Rosmarinic Acid

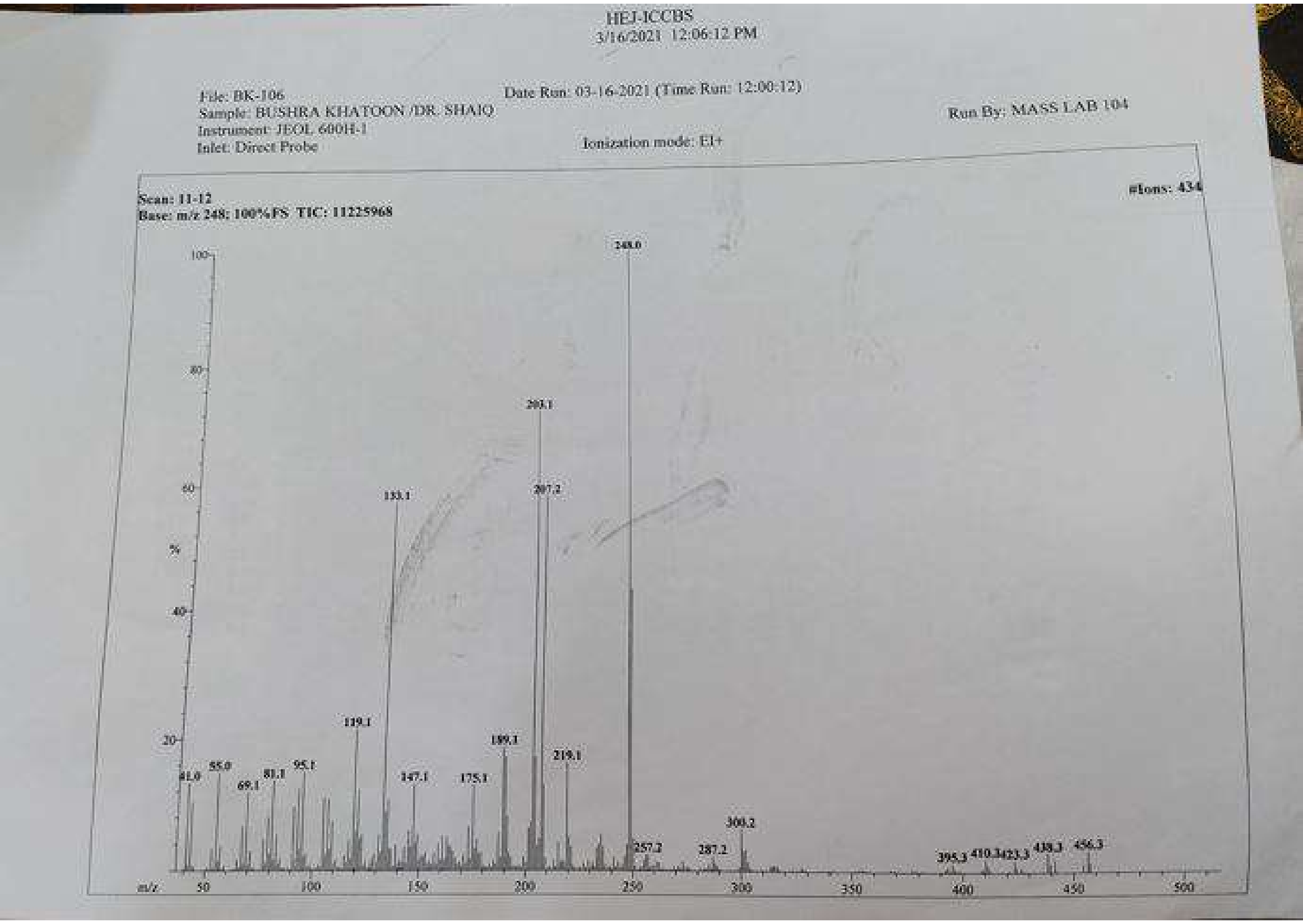

Figure S24: EIMS Spectrum of Ursolic Acid

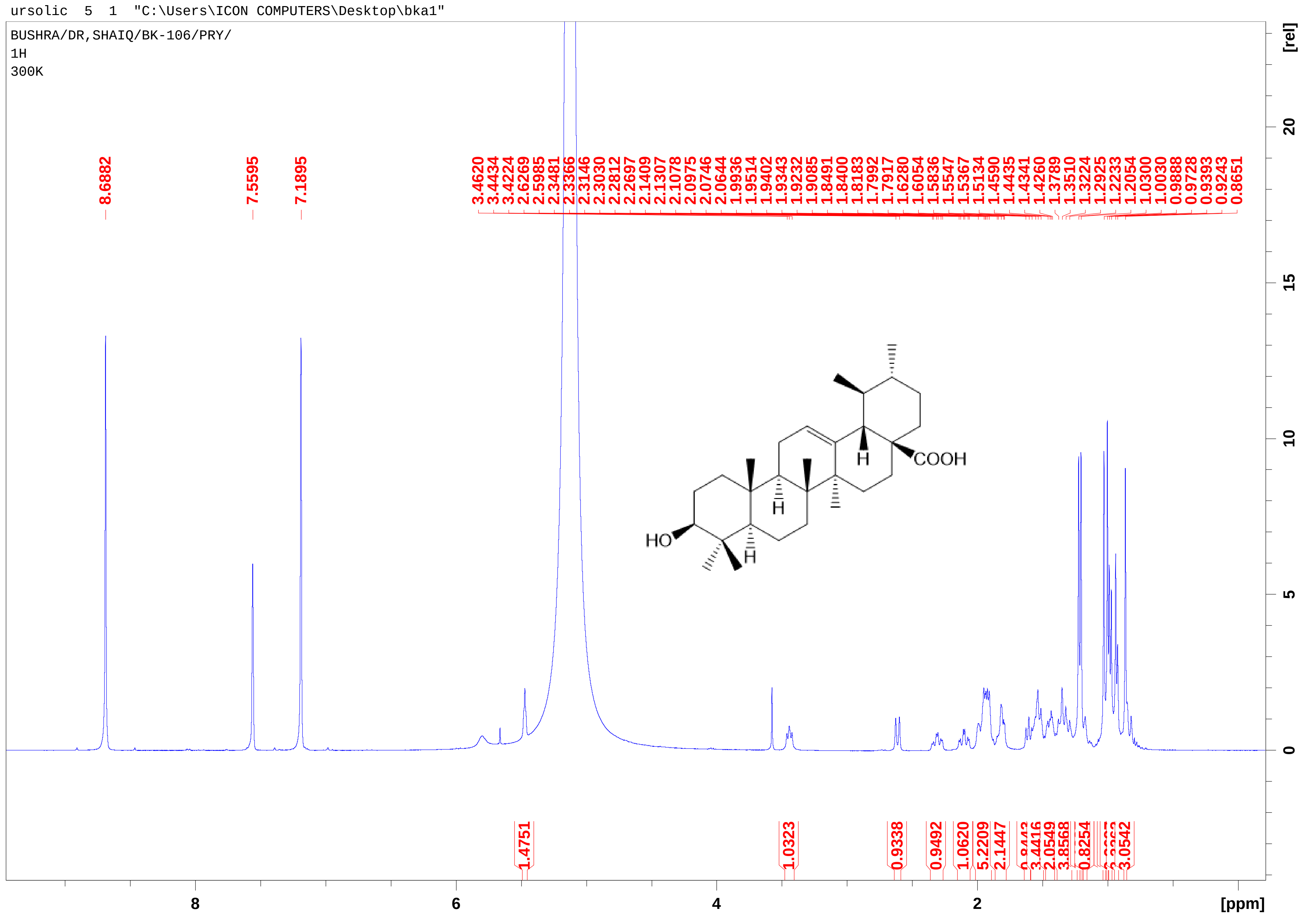

Figure S25: ¹H-NMR (C5D5N, 400 MHz) Spectrum of Ursolic Acid

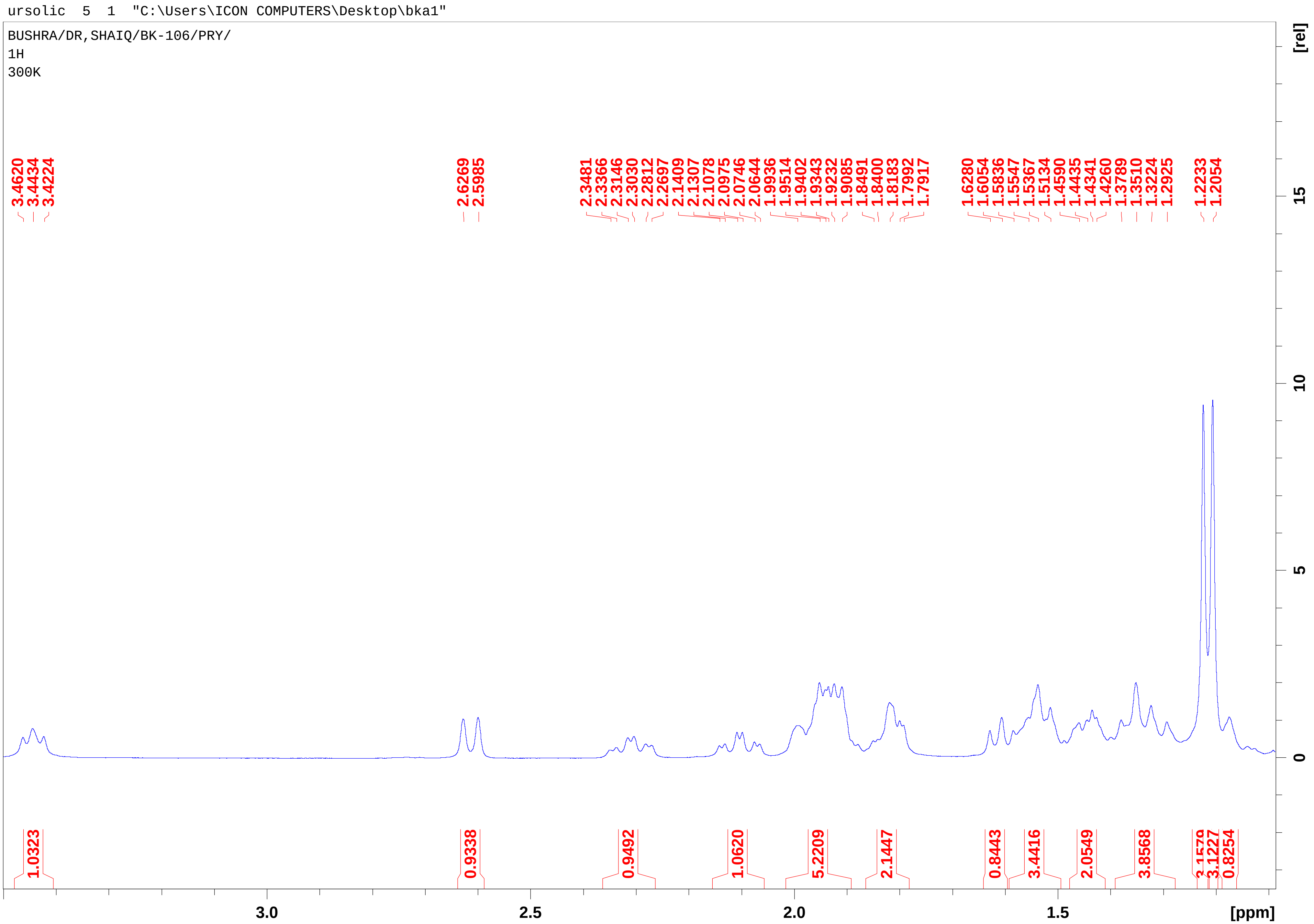

Figure S26: ¹H-NMR (C5D5N, 400 MHz) Spectrum of Ursolic Acid

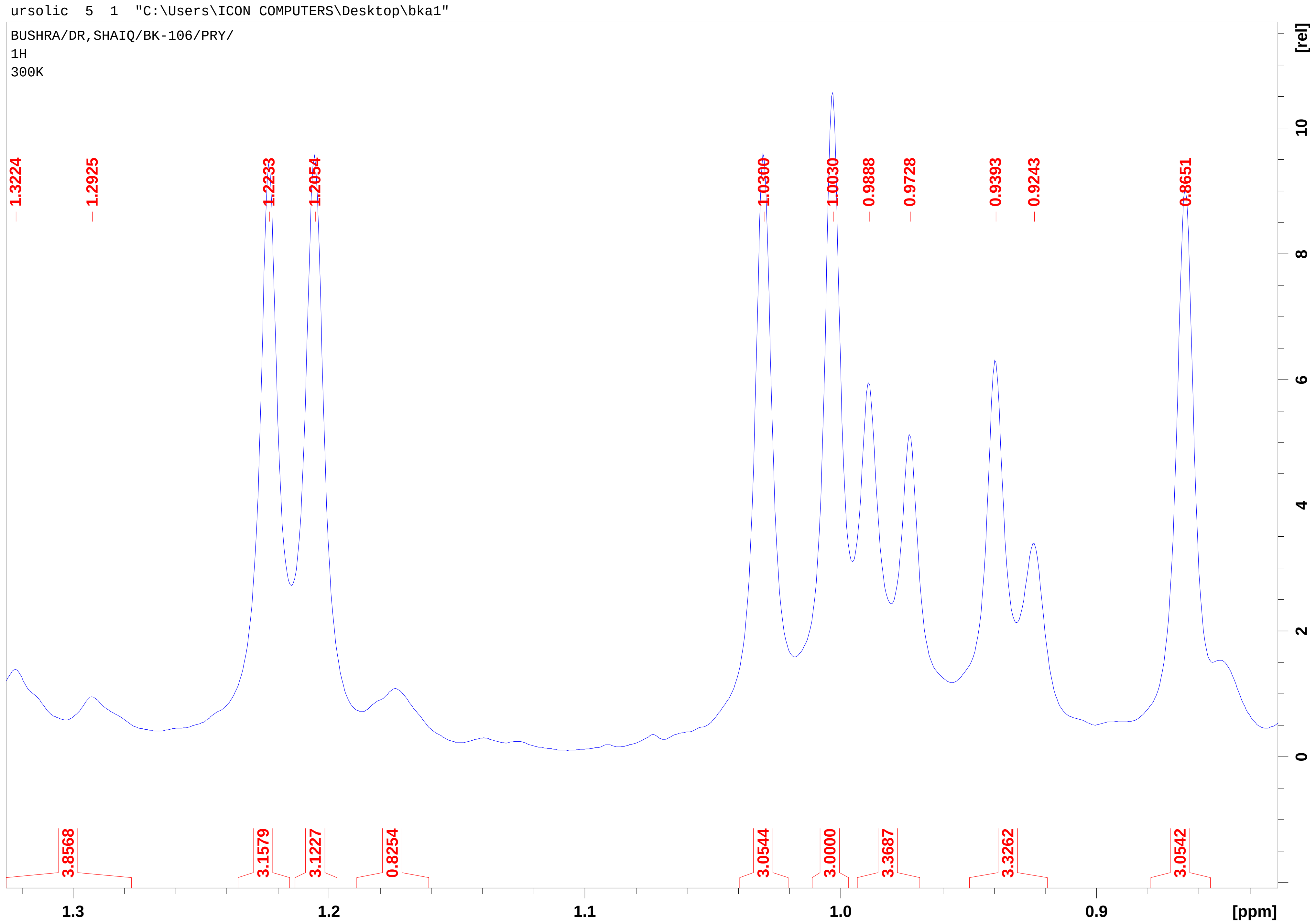

Figure S27: ¹H-NMR (C5D5N, 400 MHz) Spectrum of Ursolic Acid
